# Effect of Polyphosphoric Acid on the Coupled Aging Behavior of SBR-Modified Asphalt Under Intense UV Radiation and Large Temperature Differences

**DOI:** 10.3390/polym18182241

**Published:** 2026-09-15

**Authors:** Yanling Xu, Bo Tian, Xuejuan Cao, Junxing Wang

**Affiliations:** 1 Chongqing Municipal Facilities Safety Operation and Maintenance Application Technology Promotion Center, Chongqing City Vocational College, Chongqing 402160, China; Fengzhongdebai_he@126.com; 2 School of Civil Engineering, Chongqing Jiaotong University, Chongqing 400074, China; 3 Research Institute of Highway Ministry of Transport, Beijing 100088, China; 4 Chongqing Weishan Road Maintenance Co., Ltd., Chongqing 400014, China

**Keywords:** PPA/SBR composite modified asphalt, coupled aging, rheological properties, nanomechanical properties, gradient aging, stabilization mechanism

## Abstract

Asphalt pavements in high-altitude cold regions are subjected to the coupled effects of intense ultraviolet (UV) radiation and large temperature differences, which accelerate the oxidative aging and cracking of styrene-butadiene rubber-modified asphalt (SBR-MA), severely compromising their service durability. To improve aging resistance, polyphosphoric acid (PPA) was incorporated into SBR-MA to produce a PPA/SBR-modified binder (PPA/SBR-MA). This study systematically investigated the multiscale evolution of rheological properties, chemical structure, molecular weight distribution, nanoscale morphology, and nanomechanical properties of both SBR-MA and PPA/SBR-MA under coupled aging conditions of intense UV radiation and large temperature differences. The results indicated that macroscopic surface cracking in PPA/SBR-MA was significantly less severe than that in SBR-MA after aging. In terms of high-temperature rutting resistance and fatigue life, the PPA/SBR-MA exhibited a three-stage evolution pattern of “initial enhancement—subsequent deterioration—subsequent recovery,” while the SBR-MA showed only a two-stage pattern of “initial enhancement—subsequent deterioration.” Notably, PPA/SBR-MA consistently outperformed SBR-MA both before and throughout aging. Mechanistically, PPA reacts with polar asphaltene components to form phosphate ester linkages, thereby enhancing crosslinking between SBR and asphalt, suppressing polymer chain scission and stabilizing the colloidal structure. This retards internal asphaltene decomposition and small-molecule migration while suppressing asphaltene aggregation near the surface layer, thereby alleviating surface embrittlement. Compared with SBR-MA, the PPA/SBR-MA exhibits a 68% reduction in the surface-to-bulk modulus ratio, indicating a significantly mitigated gradient aging effect along the depth direction, which in turn suppresses macroscopic surface cracking. These findings reveal a phosphorylation-driven stabilization mechanism, offering a rational materials design paradigm for pavement applications in extreme environments.

## 1. Introduction

The service performance of asphalt pavements is severely challenged by intense ultraviolet (UV) radiation and large temperature differences in the high-altitude cold regions of western China. The Tibetan Plateau, with elevations ranging from 3000 to 5000 m, is characterized by extreme temperature differences (e.g., the extreme diurnal surface temperature difference in the Tibet Autonomous Region reaches up to 64.9 °C [1]) and the strongest UV radiation intensity in China (e.g., the average daily maximum UV radiation in Lhasa and Nagqu exceeds 72.1 W/m^2^ [2]). Such extreme climatic conditions accelerate the aging of asphalt surface materials and exacerbate early-stage distresses such as thermal cracking, which are much more severe than those observed in inland regions, ultimately leading to a significantly shortened service life of asphalt pavements [3,4]. SBR-MA has been widely used in high-altitude cold regions because of its excellent low-temperature ductility and adhesion properties [5]. SBR can form a network structure in asphalt, thereby effectively improving its resistance to external deformation [6]. However, previous studies have shown that SBR-MA pavements gradually exhibit insufficient high-temperature rutting resistance and severe aging with increasing exposure to environmental actions and vehicle loads [7,8]. Although the aging rate of SBR-MA under the coupled effects of intense UV radiation and large temperature differences is slower than that of base asphalt, surface cracks may appear earlier [9]. This phenomenon seriously restricts the engineering application of SBR-MA in the extreme environments of western China. Therefore, suppressing early cracking of SBR-MA under such extreme conditions while improving its high-temperature deformation resistance and anti-aging capability during later service has become a critical issue. Accordingly, identifying a cost-effective additive that can simultaneously enhance inherent performance and impart anti-aging ability is of significant engineering value.

To enhance the aging resistance of asphalt, researchers have explored various approaches. Inorganic fillers such as carbon black and nano-oxides can effectively shield ultraviolet (UV) radiation, but often at the expense of low-temperature performance [10,11,12]. UV absorbers are also effective; however, their practical application is limited by compatibility issues with asphalts from different crude oil sources [13]. In recent years, polyphosphoric acid (PPA), a low-cost and highly reactive asphalt modifier, has attracted considerable attention. Studies have shown that PPA chemically reacts with polar functional groups in asphalt, alters its colloidal structure, and thereby improves high-temperature performance and aging resistance [14,15]. Liu et al. [16] investigated the PPA modification mechanism based on colloidal components and found that PPA significantly increases the complex modulus of resins while also affecting the rheological behavior of aromatics and saturates. FTIR analysis further showed that the P=O and P-O bonds introduced by PPA are mainly concentrated in asphaltenes, indicating substantial intercomponent mass transfer during modification. PPA has also been incorporated into SBR-MA systems to achieve complementary performance. Liang et al. [17] found that PPA improves the thermorheological behavior and compatibility of SBR-MA, promoting a more stable SBR network. Fu et al. [18] confirmed through molecular dynamics simulations and experiments that PPA can chemically react with SBR to form a more stable crosslinked structure. Ma et al. [19] incorporated PPA into SBS/crumb rubber composite modified asphalt and observed improved polymer dispersion and a more optimized microstructure. Li et al. [20] developed a high-viscosity modified asphalt using PPA as the primary modifier and found that only 2.2 wt% PPA was required for 70# base asphalt to meet the high-viscosity specification; moreover, the chemically crosslinked structure formed by PPA remained stable and became progressively stronger under thermo-oxidative aging. However, a recent review by Liang et al. [21] noted that although PPA composite-modified asphalt exhibits excellent high-temperature performance, aging resistance, and storage stability, its low-temperature performance and adhesive properties remain insufficient. Our previous study [22] demonstrated that PPA not only inhibits UV-induced degradation of SBR molecules, thereby substantially enhancing UV aging resistance, but also improves the low-temperature cracking resistance of SBR-MA without compromising its low-temperature performance.

Despite these promising findings, most existing studies have focused on single aging factors, especially conventional thermal-oxidative aging and UV aging. By contrast, investigations into the anti-aging performance of PPA/SBR-MA under coupled intense UV radiation and large temperature differences remain scarce. In particular, systematic studies are lacking on the microstructural evolution, chemical compositional changes, and their correlation mechanisms with the macroscopic performance of PPA/SBR-MA during coupled aging.

Although the present study builds on the coupled aging platform established in our previous work [23] and extends our earlier findings on SBR-MA [9] and PPA/SBR-MA under single UV aging [22], it makes three incremental contributions. First, to the best of our knowledge, this is the first systematic parallel comparison of SBR-MA and PPA/SBR-MA under coupled intense UV and large temperature-difference aging conditions, whereas ref. [22] investigated only single UV aging. Second, macro-rheological characterization using DSR and LAS is introduced into this coupled-aging context, revealing a distinct three-stage evolution pattern for PPA/SBR-MA. Third, quantitative nanomechanical evidence is provided, showing a 68.4% reduction in the surface-to-bulk modulus ratio compared with SBR-MA, which indicates that PPA mitigates gradient aging and suppresses surface cracking under the coupled effects of intense UV radiation and large temperature differences. These aspects have not been comprehensively addressed in our previous studies.

Accordingly, this study takes PPA/SBR-MA as the research subject, with SBR-MA as a reference. Through comprehensive macro- and micro-scale testing, it systematically investigates the coupled evolution of rheological properties, chemical composition, molecular weight distribution, microstructure, and micromechanical properties under intense UV radiation and large temperature differences (i.e., the extreme diurnal surface temperature difference in the Tibetan Plateau), thereby elucidating the underlying anti-aging mechanism. The findings are expected to provide a theoretical basis and technical support for the design of long-life asphalt pavements in high-altitude cold regions.

## 2. Materials and Methods

**Figure 1:**
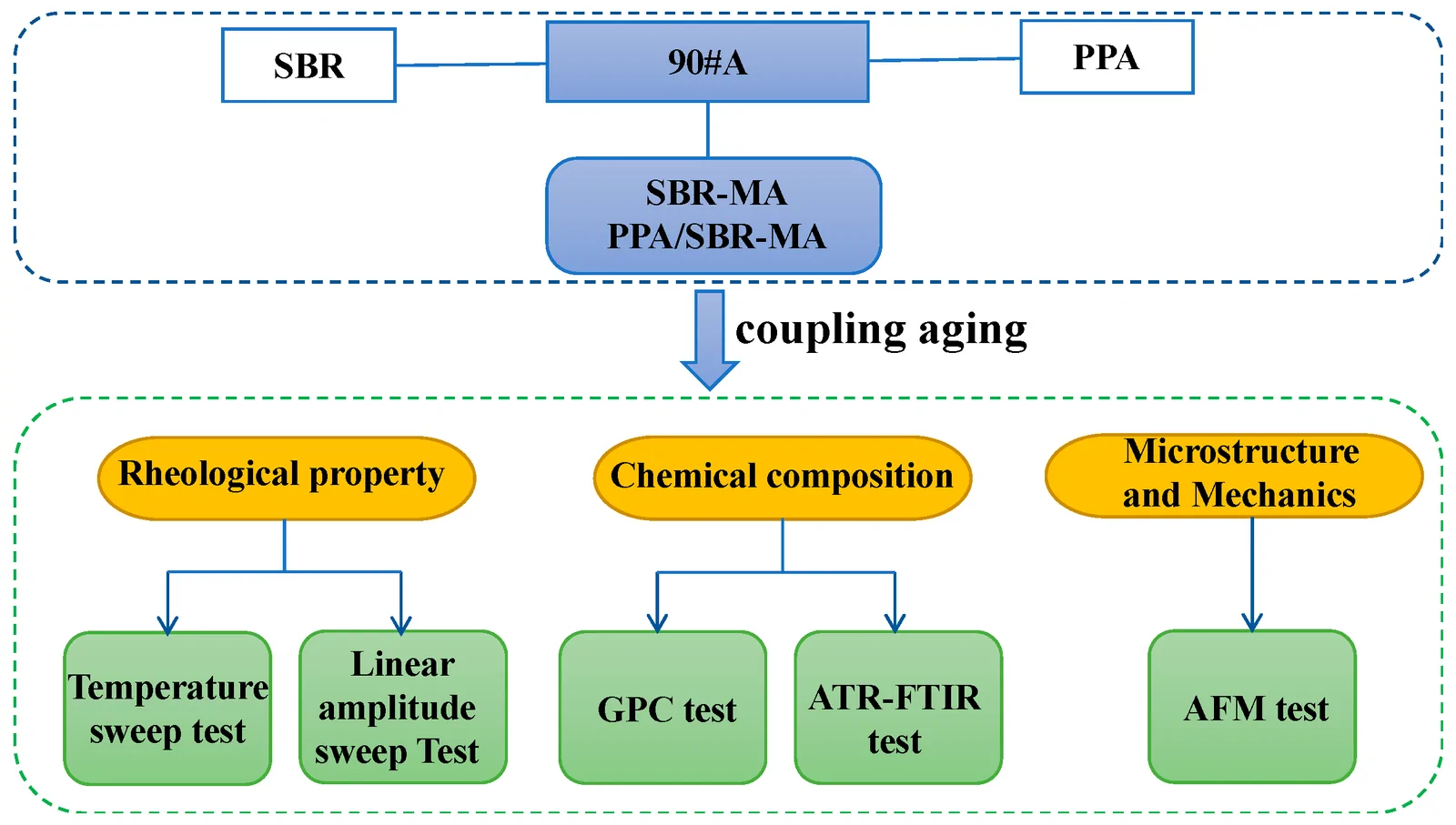
Experimental design framework.

The experimental methods used in this study are presented in Figure 1. Rheological tests were conducted to characterize the high-temperature and fatigue properties of the asphalt binder. Gel permeation chromatography (GPC), attenuated total reflectance Fourier transform infrared spectroscopy (ATR-FTIR), and atomic force microscopy (AFM) were employed to elucidate the underlying mechanisms and characterize the microscale aging effects.

### 2.1. Experimental Material

In this study, Shell 90 asphalt was used as the base binder. SBR, in the form of a white powder, was employed as the polymer modifier at a dosage of 4.0 wt% relative to the base asphalt. Industrial-grade polyphosphoric acid (PPA, H_6_P_4_O_13_, with an H_3_PO_4_ equivalent of 115%) was also incorporated. The SBR-modified asphalt samples were prepared according to the procedure described in our previous work [9]. Based on the literature and prior work by our group [18,24], the preparation procedure for PPA/SBR-MA was as follows. All modifier dosages are expressed as weight percentages relative to the base asphalt. The Shell 90 base asphalt was first dehydrated and heated to approximately 150 °C. Then, 4.0 wt% SBR was added in several portions and stirred at the same temperature for 15 min to ensure uniform dispersion before subsequent high-shear mixing. The mixture was then sheared at 4000–4500 r/min at 165 °C for 30 min. Subsequently, 1.0 wt% PPA was added for shear emulsification. The temperature was raised to and maintained at 170 °C, and shearing was continued at 4000–4500 r/min for 40 min. Finally, the mixture was mechanically stirred at 160 °C and 600–700 rpm for 30 min. The fundamental physical properties of the asphalt binders were determined in accordance with Chinese standards JTG 3410–2025 [25] and JTG F40–2004 [26]. The basic properties of the raw materials and the key physical performance indices of the asphalt binders are summarized in Table 1 and Table 2, respectively.

### 2.2. Combined Aging Test

Aging was accelerated using a custom-built coupled environmental chamber developed in our previous work [23]. Asphalt specimens were cast as films of 14 cm diameter and 1 mm thickness, and exposed to the following conditions: UV irradiation at 180 W/m^2^ on the sample surface, generated by two 1000 W high-pressure mercury lamps with a wavelength range of 315–450 nm and a peak wavelength of 365 nm; a cyclic temperature regime ranging from −20 °C to 50 °C with a 6 h period, including 2 h isothermal holds at the high and low temperature endpoints and 1 h linear ramps between them; and a constant relative humidity of 30%. The aging program ran for 20 days, with sampling at 5-day intervals, denoted as 0 d, 5 d, 10 d, 15 d, and 20 d (corresponding to days 0, 5, 10, 15, and 20) for subsequent testing, equivalent to 0, 20, 40, 60, and 80 thermal cycles, respectively.

### 2.3. Experimental Methods

#### 2.3.1. Temperature Sweep Test

Temperature sweep tests were carried out to evaluate the high-temperature rheological behavior of asphalt before and after aging. The measurements were performed using a DHR-2 dynamic shear rheometer (TA Instruments, New Castle, DE, USA) equipped with 25 mm parallel plates and a 1 mm gap, following AASHTO T315 [27]. In strain-controlled mode, a constant strain of 8% was applied, and the specimens were tested from 52 °C to 76 °C at 6 °C increments. A 10 min equilibration period was allowed at each temperature [25].

#### 2.3.2. Linear Amplitude Sweep (LAS) Test

Linear amplitude sweep (LAS) tests were conducted in accordance with AASHTO T 391-20 [28] to evaluate the fatigue resistance of asphalt binders based on the viscoelastic continuum damage (VECD) theory. The tests were carried out at 25 °C using the same DHR-2 dynamic shear rheometer (DSR; TA Instruments, New Castle, DE, USA) as described in Section 2.3.1. The tests comprised two steps: (1) a frequency sweep from 0.2 to 30 Hz at a constant strain of 0.1%. The slope m was obtained from the double-logarithmic relationship between storage modulus $G^{\prime }(\omega )=\left|G^{*}\right|(\omega )\times \cos\delta (\omega )$ and angular frequency, which was then used to calculate the damage analysis parameter α=1+1/m; (2) an amplitude sweep at 10 Hz with strain amplitudes linearly increasing from 0.1% to 30% (31 strain levels in total, each lasting 10 s). The peak shear stress, complex shear modulus ($\left|G^{*}\right|$, Pa) and phase angle (δ, degrees) are recorded at each frequency. According to the VECD theory, the damage accumulation D(t) is calculated by Equation (1). The damage characteristic curve was fitted using the power-law model shown in Equation (2):

(1) $$D\left(t\right)≅{\sum_{i=1}^{N}\left[\pi I_{D}\gamma_{0}^{2}(\left|G^{*}\right|\sin\delta_{i-1}-\left|G^{*}\right|\sin\delta_{i})\right]}^{\frac{\alpha }{1+\alpha }}{\left(t_{i}-t_{i-1}\right)}^{\frac{1}{1+\alpha }}$$

where $I_{D}$ is the initial value of $\left|G^{*}\right|$ from the 1.0% applied strain interval (MPa); $\gamma_{0}$ is the applied strain for a given data point (%); $\left|G^{*}\right|$ is the complex shear modulus (MPa); and t is the testing time (s).

(2) $$\left|G^{*}\right|\sin\delta =C_{0}-C_{1}{\left(D\right)}^{C_{2}}$$

where $C_{0}$ is the average value of $\left|G^{*}\right|\sin\delta$ from the 0.1% strain interval; and $C_{1}$ and $C_{2}$ are curve-fit coefficients derived through linearization of the power law.

The parameters $C_{1}$ and $C_{2}$ were determined by linear regression of the following Equation (3):

(3) $$\log(C_{0}-\left|G^{*}\right|\cdot \sin\delta )=\log(C_{1})+C_{2}\cdot \log(D)$$

where $C_{1}$ is calculated as the antilog of the intercept and $C_{2}$ is calculated as the slope of line formed as $\log\left(C_{0}-|G^{*}|\cdot \sin\delta \right)$ versus $log\left(D\right)$.

The failure damage value $D_{f}$ is defined as the damage value corresponding to a 35% reduction in the undamaged $\left|G^{*}\right|\sin\delta$, the calculation is calculated by Equation (4);

(4) $$D_{f}=(0.35){\left(\frac{C_{0}}{C_{1}}\right)}^{\left(\frac{1}{C_{2}}\right)}$$

The fatigue parameters $A_{35}$ and B are calculated by Equation (5)

(5) $$A_{35}=\frac{f{\left(D_{f}\right)}^{k}}{k(\pi C_{1}C_{2})\alpha },B=2\alpha$$

where f is the loading frequency (10 Hz), and $k=1+\left(1-C_{2}\right)\alpha$. The fatigue life of the asphalt binder is calculated by Equation (6):

(6) $$N_{f}=A_{35}{\left(\gamma_{\max}\right)}^{B}$$

where $N_{f}$ is the fatigue life of the asphalt binder (cycles); $\gamma_{\max}$ is the estimated maximum binder strain (%) in the pavement structure.

#### 2.3.3. ATR-FTIR Test

To investigate the compact thin film formed on the asphalt surface under the combined effects of intense UV irradiation and large temperature differences, as well as the differential aging behavior between the surface layer and the bulk of PPA/SBR-MA, ATR-FTIR (Thermo Fisher IS50, Thermo Fisher, Waltham, MA, USA) was used for in situ, non-destructive characterization. The samples were placed directly on a multiple-reflection horizontal ZnSe ATR crystal. Spectra were collected over the range of 400 to 4000 cm^−1^ at a resolution of 1 cm^−1^, with 32 scans accumulated.

The extent of aging was evaluated using functional group indices (I), defined as the ratio of the characteristic peak area to the total integrated area (450–4000 cm^−1^). The specific indices ($I_{CH=CH}$, $I_{S=O}$, $I_{C=C}$, $I_{C=O}$) were calculated using Equations (7)–(10) [23,29].

(7) $$I_{CH=CH}=\frac{A_{966{\mathrm{cm}}^{-1}}}{\sum A_{450{\mathrm{cm}}^{-1}–4000{\mathrm{cm}}^{-1}}}$$

(8) $$I_{S=O}=\frac{A_{1030{\mathrm{cm}}^{-1}}}{\sum A_{450{\mathrm{cm}}^{-1}–4000{\mathrm{cm}}^{-1}}}$$

(9) $$I_{C=C}=\frac{A_{1600{\mathrm{cm}}^{-1}}}{\sum A_{450{\mathrm{cm}}^{-1}–4000{\mathrm{cm}}^{-1}}}$$

(10) $$I_{C=O}=\frac{A_{1700{\mathrm{cm}}^{-1}}}{\sum A_{450{\mathrm{cm}}^{-1}–4000{\mathrm{cm}}^{-1}}}$$

The definitions of the parameters in the equations are summarized in Table 3.

#### 2.3.4. GPC Test

An Agilent 1260 GPC system (Agilent Technologies, Waldbronn, Germany) was used to characterize asphalt binders before and after aging. Approximately 10 mg of each sample was dissolved in 5 mL of chromatographic-grade THF and allowed to stand for 30 min to ensure complete dissolution. The solution was filtered through a 0.22 μm filter before injection. The separation was performed at 1 mL/min and 30 °C for 30 min, with polystyrene as the calibration standard. The GPC data were analyzed following our previous procedure [7]. The chromatogram was divided into 13 equal-time slices, with the first 5, middle 5, and last 4 corresponding to the LMS, MMS, and SMS fractions, respectively [30,31,32]. Molecular weight distribution was characterized by $M_{n}$, $\;M_{w}$, and polydispersity index (PDI = $M_{w}/M_{n}$), calculated as described in refs. [9,33]. 

#### 2.3.5. AFM Test

AFM (5500, Keysight Technologies, Santa Rosa, CA, USA) was employed to characterize the microstructure and mechanical properties of asphalt binders. Morphological imaging was performed in tapping mode using an HQ: NSC14/Pt probe (MikroMasch, Tallinn, Estonia; spring constant: 5.0 N/m, resonant frequency: 160 Hz) over a 20 × 20 μm^2^ area at a scan rate of 0.5 Hz and a resolution of 256 × 256 pixels. Force curve measurements were conducted in contact mode using a PPP-CONT-20 probe (NanoSensors, Neuchâtel, Switzerland; spring constant: 0.02–0.77 N/m, resonant frequency: 6–21 Hz) at a ramp rate of 0.5 μm/s. The probe stiffness and cantilever deflection sensitivity were calibrated prior to testing. Six replicate measurements were performed for each sample at 25 °C. Data were analyzed using Pico Image Elements 1.18 and Gwyddion 2.57 software [23]. Micro-mechanical properties, including adhesion force and Young’s modulus based on the DMT model, were determined following the same procedure described in our previous studies [7,23].

## 3. Results and Discussions

### 3.1. Macroscopic Morphology Analysis

**Figure 2:**
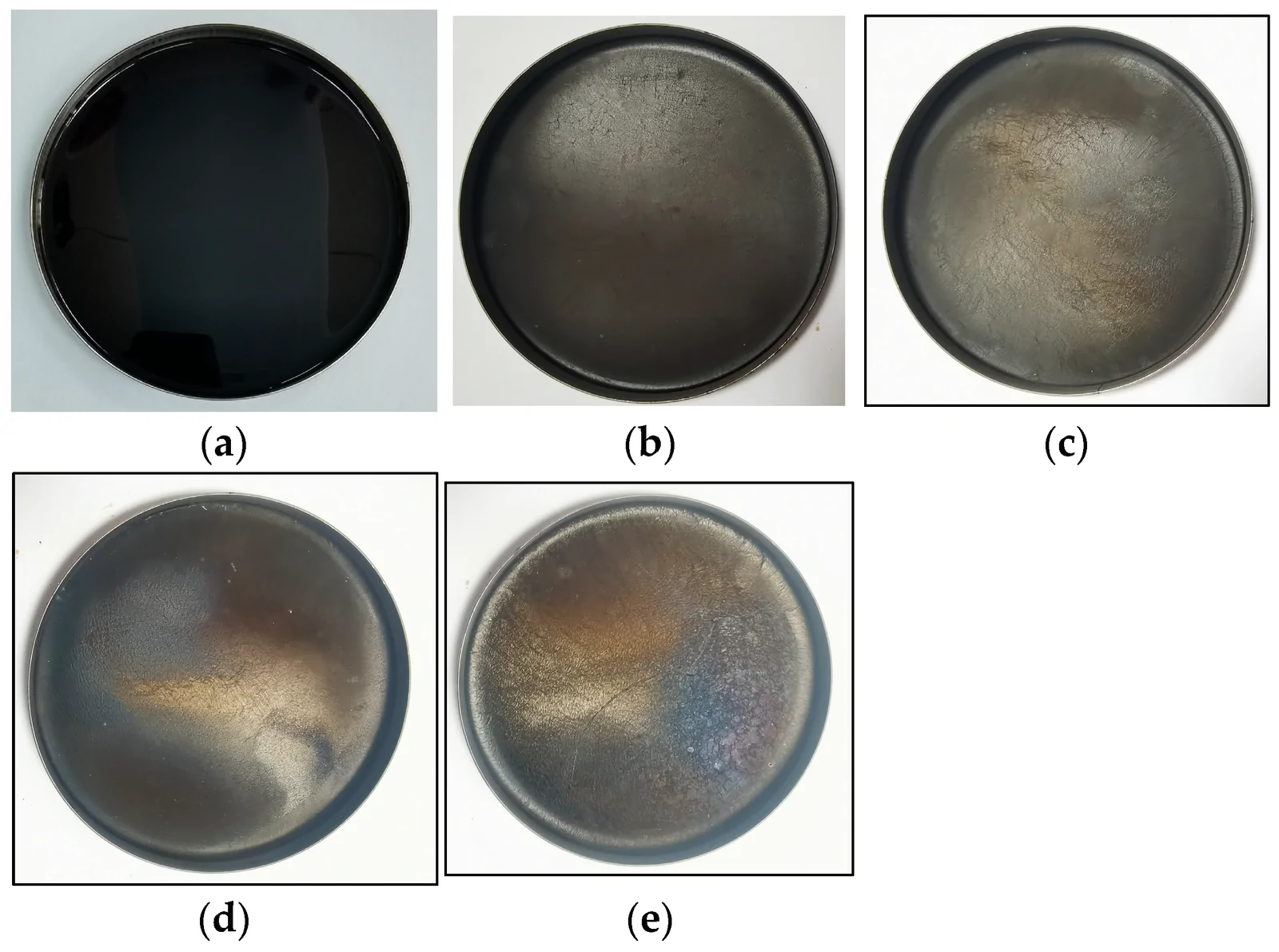
Surface appearance evolution of PPA/SBR-MA with aging time: (**a**) 0 d, (**b**) 5 d, (**c**) 10 d, (**d**) 15 d, and (**e**) 20 d.

As shown in Figure 2, with the progression of aging under the combined effects of UV radiation and large temperature differences, significant surface deterioration occurred in the asphalt specimens. Compared with the SBR-MA reported in the literature [9], PPA/SBR-MA exhibited superior aging resistance in terms of both color evolution and surface cracking. In terms of color evolution, PPA/SBR-MA displayed a relatively gradual darkening process: it remained deep black at 0 d, gradually turned light brown at 5 d and 10 d, shifted to bluish-green at 15 d, and finally presented localized dark cyan at 20 d. Notably, its color evolution proceeded in clearly defined stages, and aging-induced discoloration occurred relatively late. In contrast, SBR-MA exhibited much more drastic color changes. Large areas of bluish-green oxidation layers appeared on the material surface during the middle-to-late aging stage (15 d), and heterogeneous colors, including light yellow and dark cyan, emerged earlier with greater contrast. Similar differences were observed in surface cracking behavior. Cracks in PPA/SBR-MA gradually evolved from fine reticulated patterns into through-going cracks, indicating relatively mild structural degradation during aging. By comparison, SBR-MA rapidly developed large through-going cracks from the initial reticulated pattern, with obvious structural damage observed as early as 15 d, indicating a more abrupt and severe degradation process.

Overall, PPA/SBR-MA underwent a milder and more staged aging process, whereas SBR-MA suffered more intense oxidation and severe structural deterioration. These results highlight a contrast between gradual and abrupt failure mechanisms in the two modified asphalt systems.

### 3.2. High Temperature Rheological Properties Analysis

With prolonged environmental exposure and repeated traffic loading, SBR-MA pavements have exhibited insufficient resistance to high-temperature permanent deformation and pronounced aging [7,8]. Accordingly, this study examines the effect of PPA on the high-temperature rutting resistance and fatigue resistance of SBR-MA under coupled aging conditions. In this section, the rutting factor (G*/sinδ) was used to evaluate high-temperature performance, and the evolution of this parameter with aging time was compared between PPA/SBR-MA and SBR-MA to assess aging resistance.

**Figure 3:**
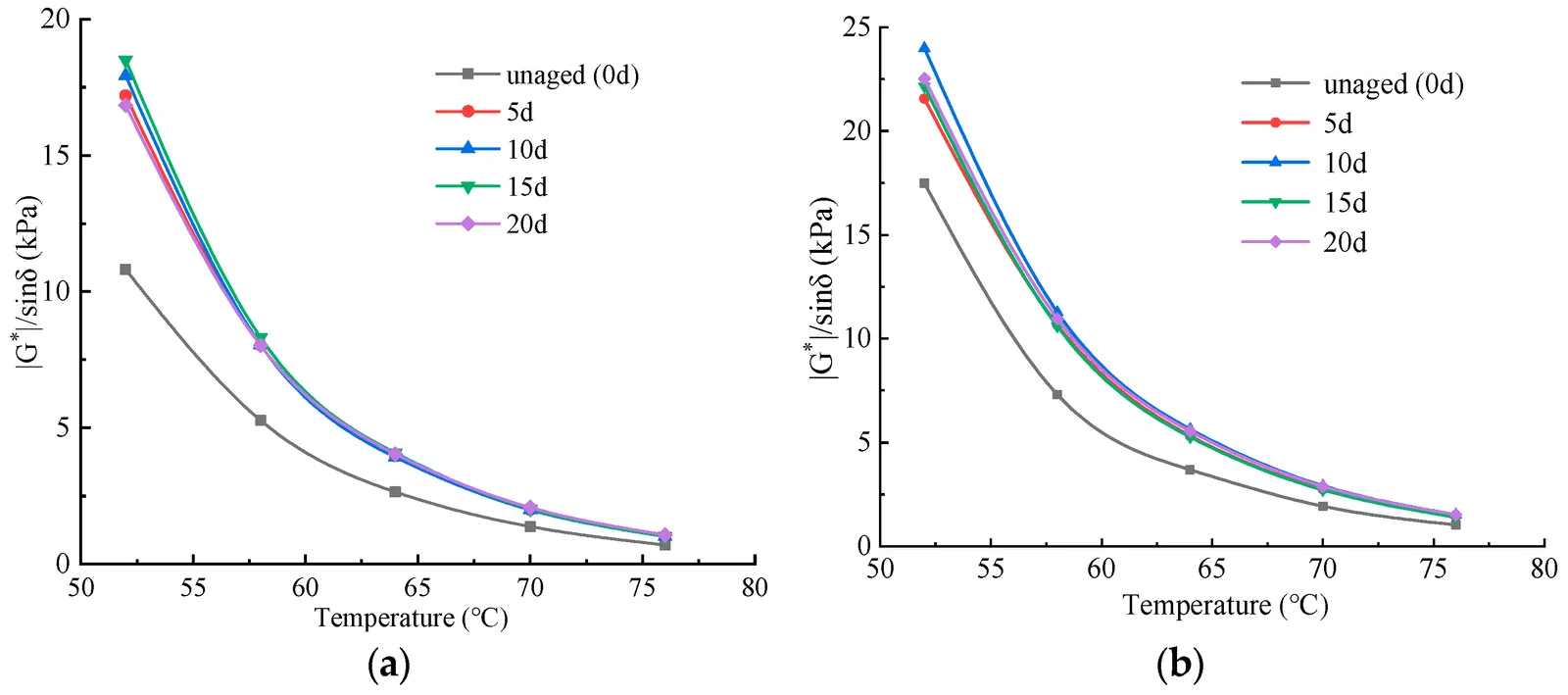
Rutting factor (|G*|/sinδ) curves of (**a**) SBR-MA and (**b**) PPA/SBR-MA at different aging durations.

Figure 3 shows the evolution of the rutting factor for SBR-MA and PPA/SBR-MA during high-temperature frequency sweep testing under the coupled aging conditions. Overall, the addition of PPA shifts the rutting factor curve upward across the entire temperature range, indicating a significant enhancement in high-temperature permanent deformation resistance. For both asphalt binders, the rutting factor decreases monotonically with increasing temperature. The decline is steeper below 70 °C and more gradual above 70 °C, reflecting reduced sensitivity of the viscoelastic response in the high-temperature regime.

As aging progresses, SBR-MA exhibits a typical “strengthening-then-deterioration” trend: the rutting factor continuously rises during the early aging stage (0–10 d), peaks at 15 d, and then declines noticeably at 20 d, indicating irreversible degradation of high-temperature stability in the late stage of long-term coupled aging. In contrast, PPA/SBR-MA displays a distinctive stage-wise non-monotonic evolution: the rutting factor rises from 0 to 10 d, briefly declines at 15 d, and then rises again at 20 d. Notably, its rutting factor remains above its initial value throughout aging, demonstrating superior high-temperature stability. This late-stage performance recovery suggests that PPA effectively delays performance degradation and enhances resistance to permanent deformation under prolonged harsh exposure, thereby overcoming the deficiencies of conventional SBR-MA in high-temperature rutting resistance and long-term aging resistance.

**Figure 4:**
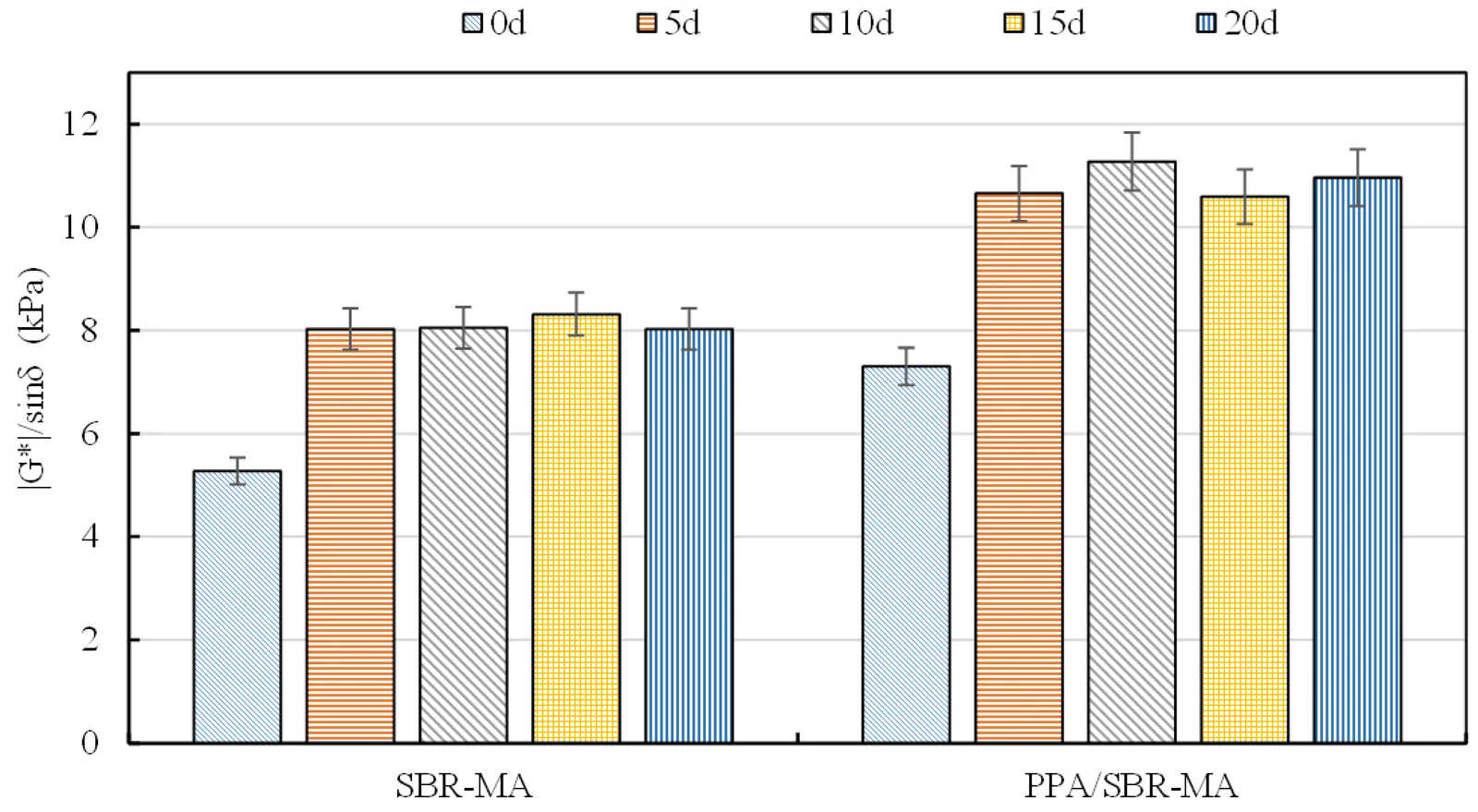
Rutting factor ($\left|G^{*}\right|$/sinδ) of asphalt aged at 58 °C for different durations.

To further quantify the aging-induced performance evolution, comparative analysis was conducted at 58 °C, a widely adopted temperature for high-temperature evaluation, as shown in Figure 4. For SBR-MA, the rutting factor increases steadily from 0 d to 15 d, peaks at 15 d with a value approximately 52.24% higher than that at 0 d, and then declines at 20 d, exhibiting a unimodal decay trend. This trend is highly consistent with the statistical finding that severe rutting distress frequently occurs in the later service stage of pavements in high-altitude cold regions [34], reflecting the detrimental effect of the late-stage high-temperature performance deterioration of SBR-MA on the actual rutting resistance of the pavement. In contrast, PPA/SBR-MA exhibits a bimodal evolution: a rapid increase from 0 d to 10 d, a slight decline at 15 d while remaining higher than the initial value, and a rebound at 20 d with a value approximately 50.10% higher than that at 0 d. These results indicate that PPA not only delays the attenuation turning point of high-temperature performance but also triggers a performance re-enhancement mechanism in the later aging stage, thereby significantly improving the material’s adaptability and durability under prolonged harsh environmental conditions. In summary, from the perspective of macroscopic high-temperature performance response, PPA/SBR-MA systematically outperforms SBR-MA in terms of high-temperature rutting resistance and anti-aging durability under the coupled effects of intense UV radiation and large temperature differences.

### 3.3. Fatigue Performance Analysis

Fatigue performance is a critical criterion for assessing the long-term serviceability of asphalt materials and is directly related to the service life of asphalt pavements. In this section, the linear amplitude sweep (LAS) test was used to evaluate the fatigue behavior of PPA/SBR-MA under different coupled aging durations, thereby investigating the effect of PPA on the fatigue performance of SBR-MA.

#### 3.3.1. Stress–Strain Response Analysis

**Figure 5:**
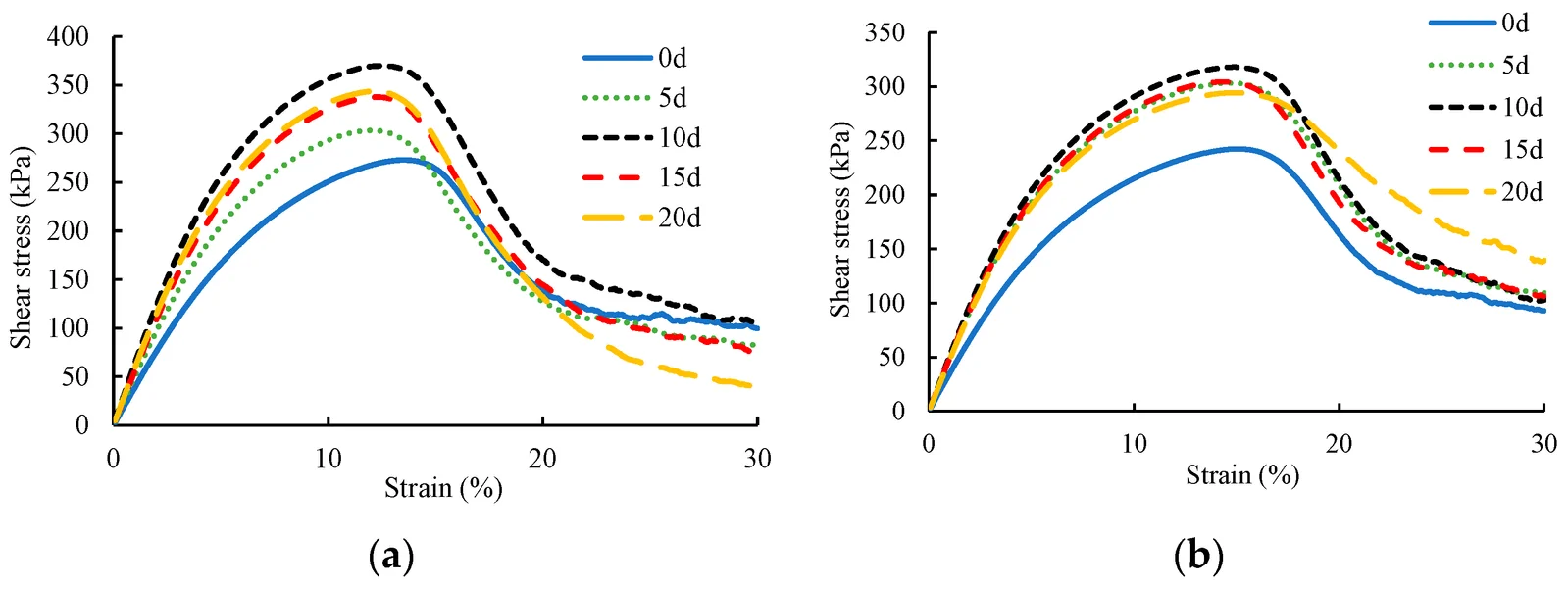
Stress–strain curves of (**a**) SBRMA and (**b**) PPA/SBR-MA at different aging durations.

During the LAS test in amplitude-sweep mode, the stress–strain curves of the asphalt binders were obtained. The stress–strain curves of SBR-MA and PPA/SBR-MA under the coupled aging conditions are shown in Figure 5.

As shown in Figure 5, the incorporation of PPA into SBR-MA significantly broadens the peak region of the stress–strain curve, resulting in a flatter peak profile. A wider peak region indicates lower strain sensitivity of the asphalt under a given shear loading rate, suggesting improved fatigue resistance. This flattened post-peak behavior may be attributed to the reinforced internal network structure induced by the polymer modifier, which enables the material to retain a certain load-bearing capacity even beyond the peak stress. The more pronounced the post-peak flattening, the more extended the descending branch, and thus the better the resistance to fatigue cracking at high strain levels [35]. From a mechanical perspective, at the same strain level, a lower peak fatigue stress implies a smaller driving force for damage accumulation and thus a slower rate of fatigue damage progression. Conversely, a higher peak fatigue strain indicates greater ultimate deformability of the asphalt material. As summarized in Table 4, the incorporation of PPA reduces peak stress while increasing peak strain, indicating enhanced toughness and improved stress–strain capacity under repeated loading.

The evolution of the stress–strain curves of PPA/SBR-MA with aging time follows a characteristic pattern: an initial upward shift followed by a subsequent downward shift. Moreover, as evident from the figure, the post-peak stress decay slope of SBR-MA across all aging durations is consistently steeper than that of PPA/SBR-MA. For instance, after 20 days of aging, SBR-MA reaches a peak stress of 309.8 kPa and then rapidly declines, exhibiting relatively brittle failure behavior. In contrast, PPA/SBR-MA attains a slightly lower peak stress of 294.4 kPa but maintains a comparatively high load-carrying capacity thereafter, with a much gentler descending slope, demonstrating favorable ductile failure characteristics. This suggests that PPA/SBR-MA possesses superior deformation capacity and energy absorption capability under impact loading. Furthermore, the peak width of the stress–strain curve for PPA/SBR-MA aged for 20 days is broader than that observed at other aging durations, implying that its fatigue resistance actually improves during the later stages of aging. By contrast, the fatigue performance of SBR-MA deteriorates markedly in the late aging stage, particularly at large strains (e.g., strain > 20%). Across all aging durations, the peak stress of PPA/SBR-MA remains lower than that of SBR-MA at the corresponding aging state, while its peak strain remains higher, indicating that PPA/SBR-MA consistently exhibits superior fatigue resistance throughout the entire aging process. Collectively, these findings demonstrate that the incorporation of PPA inhibits the aging progression of SBR-MA.

Based on the VECD model specified in AASHTO T 391-20 [27], fatigue damage curves were constructed for the asphalt samples. In these curves, the abscissa D represents the cumulative damage parameter, while the ordinate C denotes the ratio of $\left|G^{*}\right|\sin\delta$ at any time t to its initial value, serving as an indicator of the structural integrity of the asphalt material [36,37]. The fatigue damage curve reflects the evolution of the loss modulus with damage progression. Specifically, at a given cumulative damage parameter C, a smaller D indicates a stronger damage resistance of the material.

**Figure 6:**
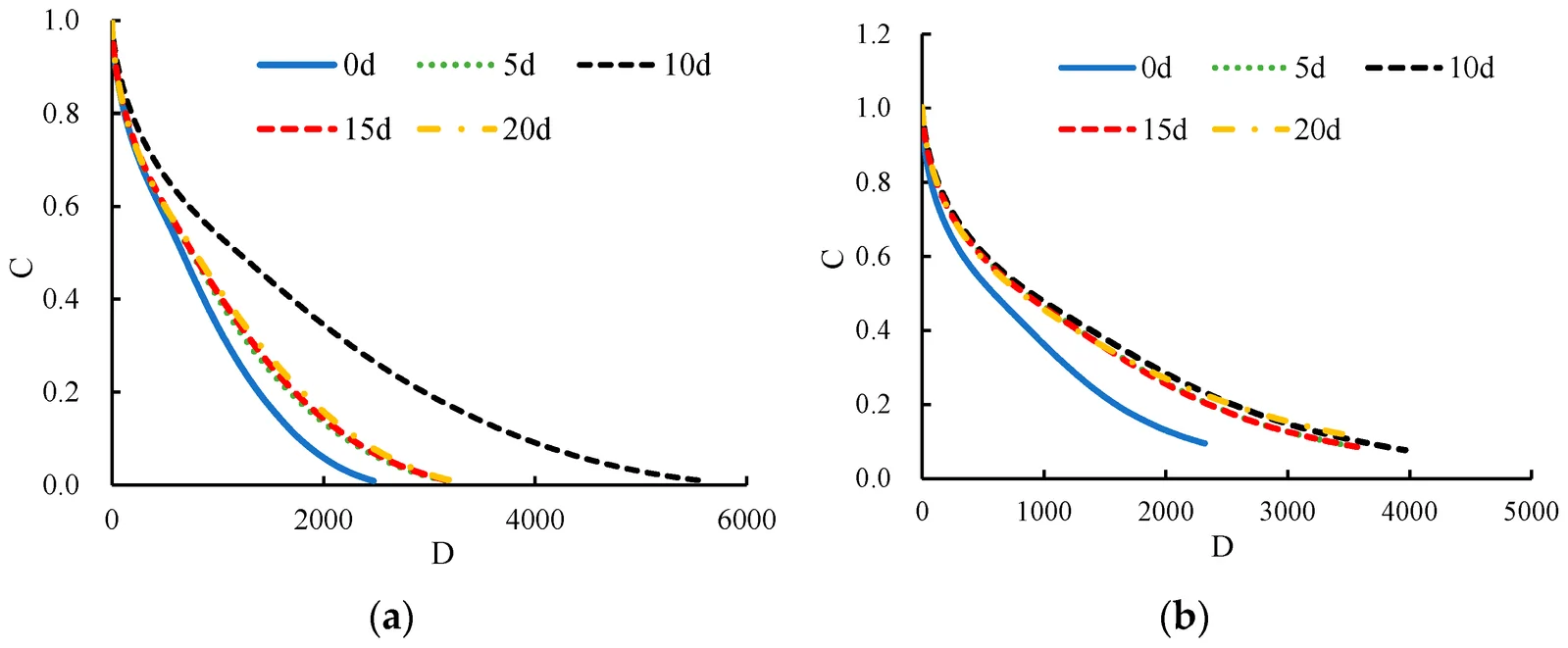
Fatigue damage curves of (**a**) SBR-MA and (**b**) PPA/SBR-MA at different aging durations.

Figure 6 presents the damage characteristic curves of SBR-MA and PPA/SBR-MA. Compared with SBR-MA, PPA/SBR-MA consistently exhibits a higher structural integrity parameter and a narrower range of D values, indicating superior damage resistance. During aging, the differences in the damage characteristic curves at various aging durations are relatively small, particularly at low strain levels, suggesting that PPA incorporation reduces the sensitivity of asphalt to the coupled effects of intense UV radiation and large temperature differences. At higher strain levels, the evolution trend of the damage curves with aging time becomes more pronounced: the curves first shift upward, then downward, and finally upward again, a pattern consistent with that of SBR-MA, but with much smaller magnitude. Overall, the reduced magnitude of curve shifts with aging time demonstrates that PPA modification effectively enhances the performance stability of asphalt under coupled aging conditions.

#### 3.3.2. Fatigue Life Analysis

**Figure 7:**
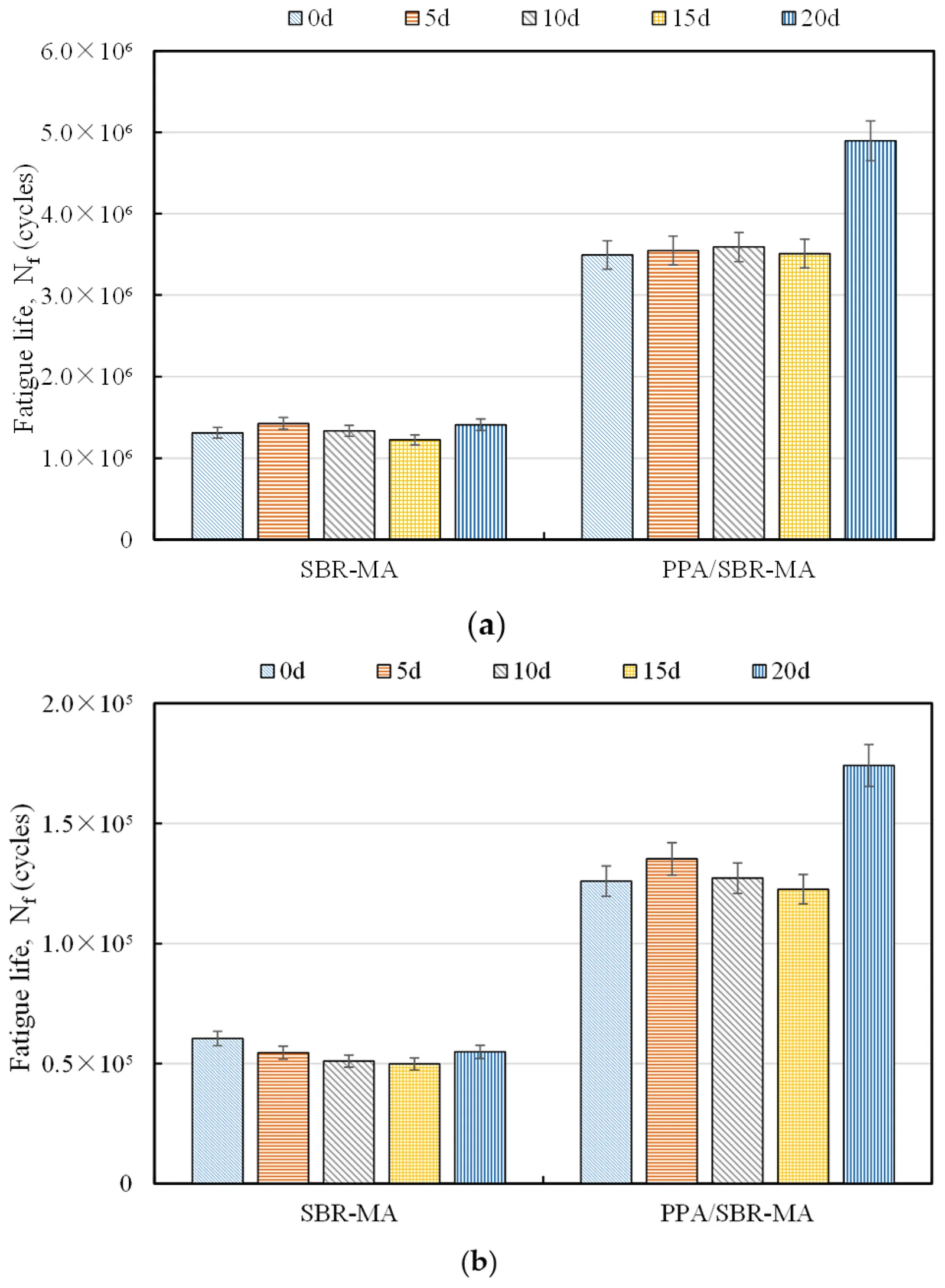
Fatigue life of SBR-MA and PPA/SBR-MA at strain levels of (**a**) 2.5% and (**b**) 5.0%.

Figure 7 presents the fatigue life results for SBR-MA and PPA/SBR-MA under the combined aging of intense UV radiation and large temperature differences, at strain levels of 2.5% and 5.0%. Under identical strain conditions, the fatigue life of PPA/SBR-MA consistently exceeds that of SBR-MA across the entire aging period, demonstrating that the incorporation of PPA effectively enhances the fatigue cracking resistance of the asphalt material. As coupling aging time increases, the fatigue life of SBR-MA exhibits only minor fluctuations without a sustained degradation trend. In contrast, the fatigue life of PPA/SBR-MA follows a three-stage evolution pattern: increase → decrease → re-increase. This non-monotonic behavior arises from the dynamic competition between molecular crosslinking and molecular chain scission during aging and is corroborated by subsequent microscopic characterization.

The test results also reveal that increasing the applied strain from 2.5% to 5.0% reduces the fatigue life of both asphalts by nearly one order of magnitude, confirming the high sensitivity of asphalt fatigue life to strain amplitude. After 20 days of aging, PPA/SBR-MA shows the highest fatigue life, and the performance gap between PPA/SBR-MA and SBR-MA further widens, indicating that PPA/SBR-MA exhibits improved long-term fatigue durability under such harsh environmental conditions.

### 3.4. Functional Group Analysis

**Figure 8:**
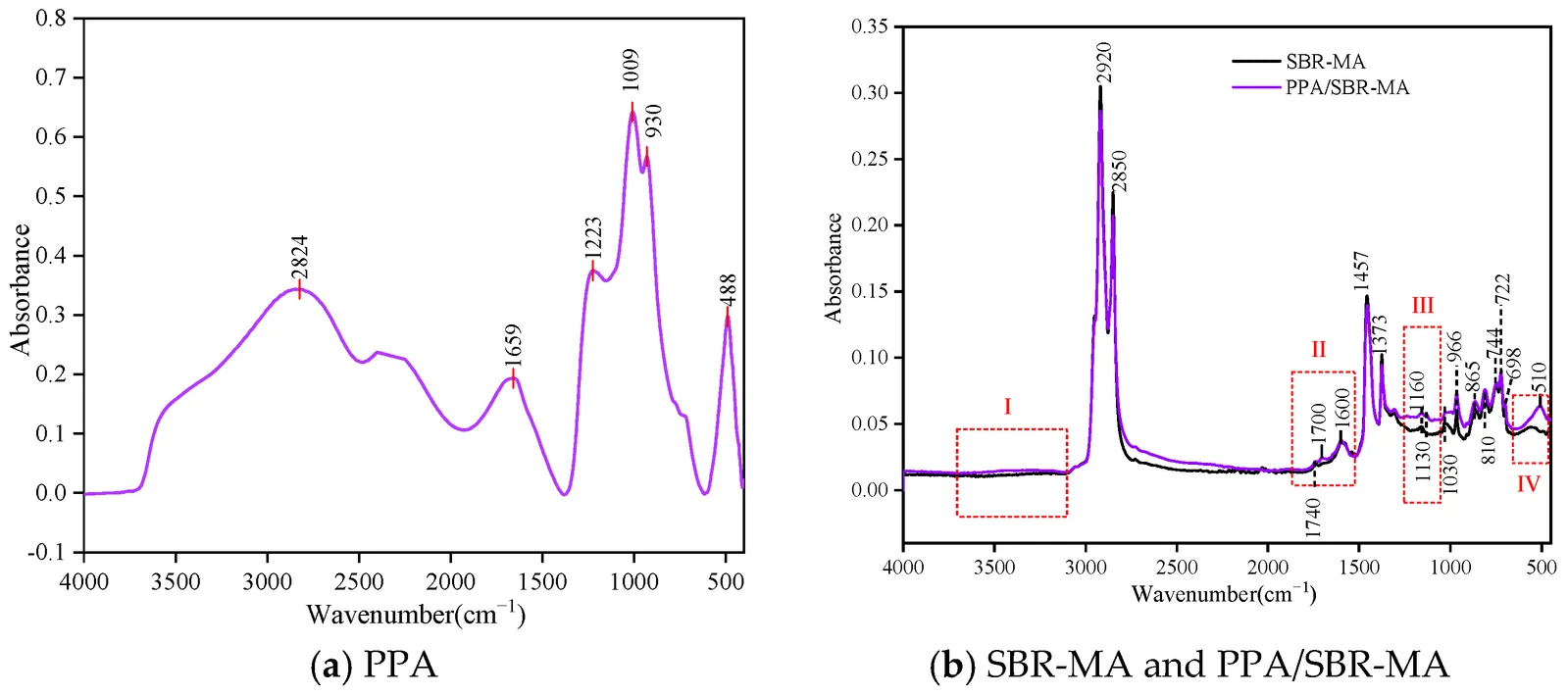
FTIR spectra of (**a**) PPA and (**b**) SBR-MA and PPA/SBR-MA.

**Figure 9:**
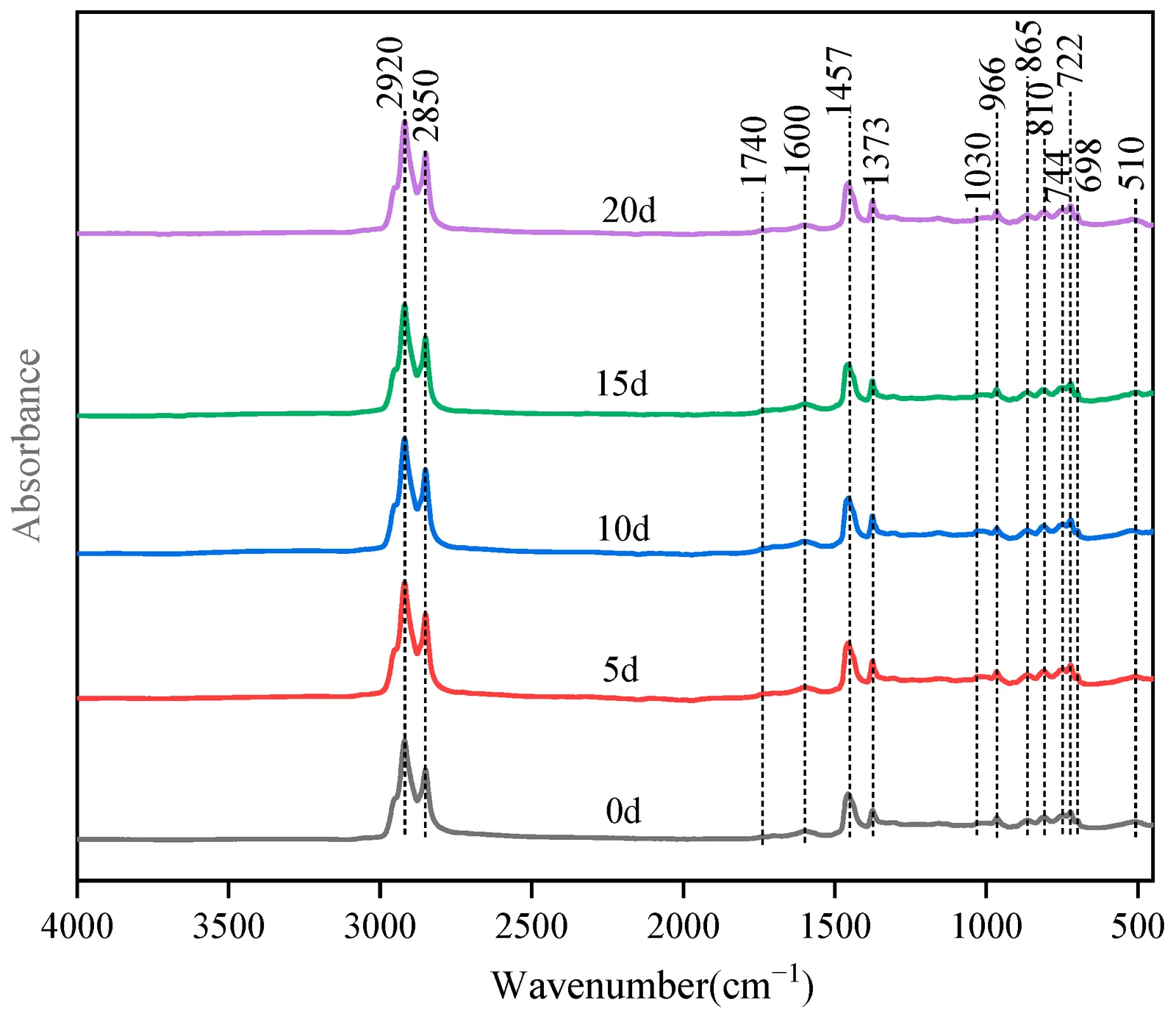
Infrared spectra of the upper layer of PPA/SBR-MA at different aging durations.

**Figure 10:**
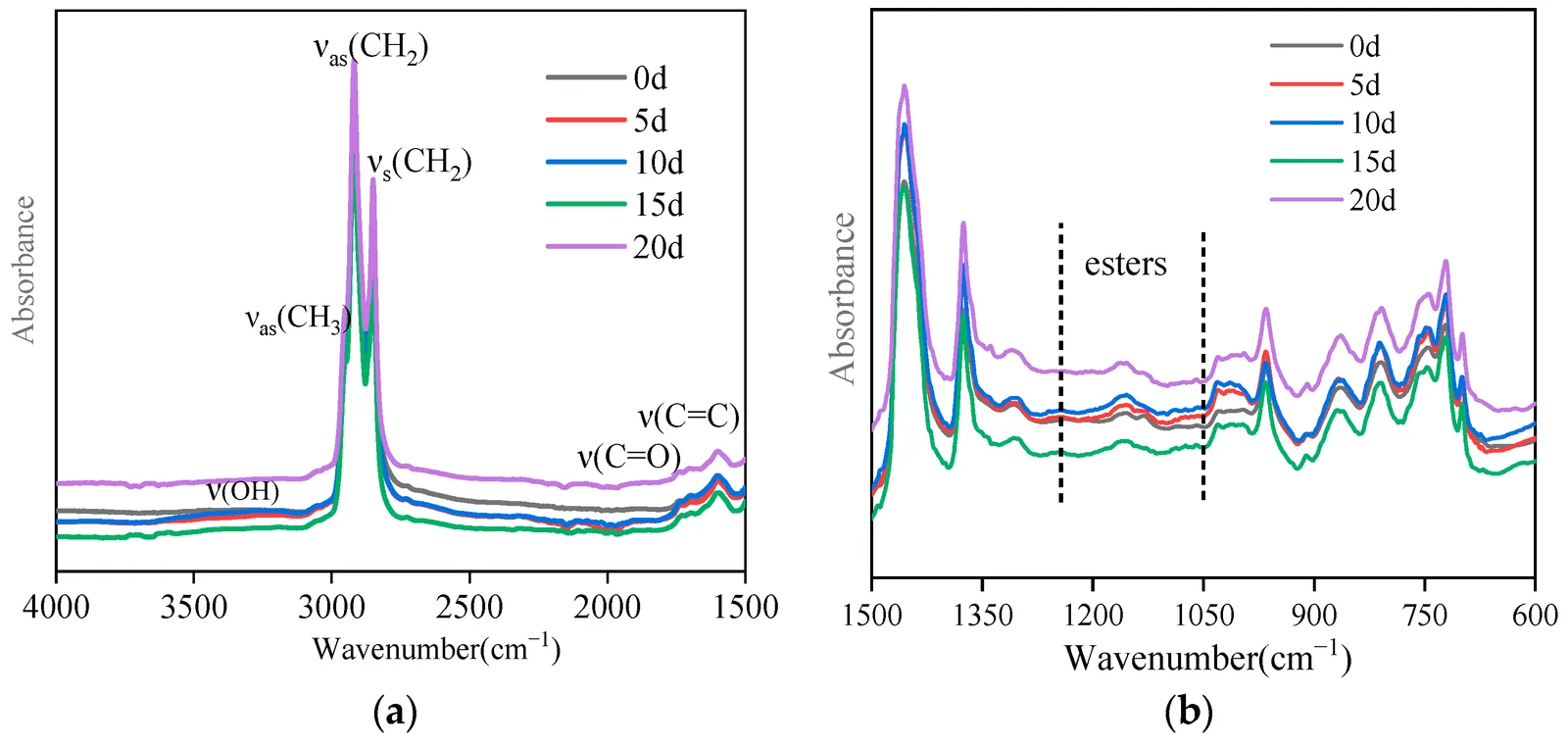
Magnified FTIR spectra of PPA/SBR-MA upper layer at different aging durations: (**a**) 4000–1500 cm^−1^ and (**b**) 1500–600 cm^−1^.

**Figure 11:**
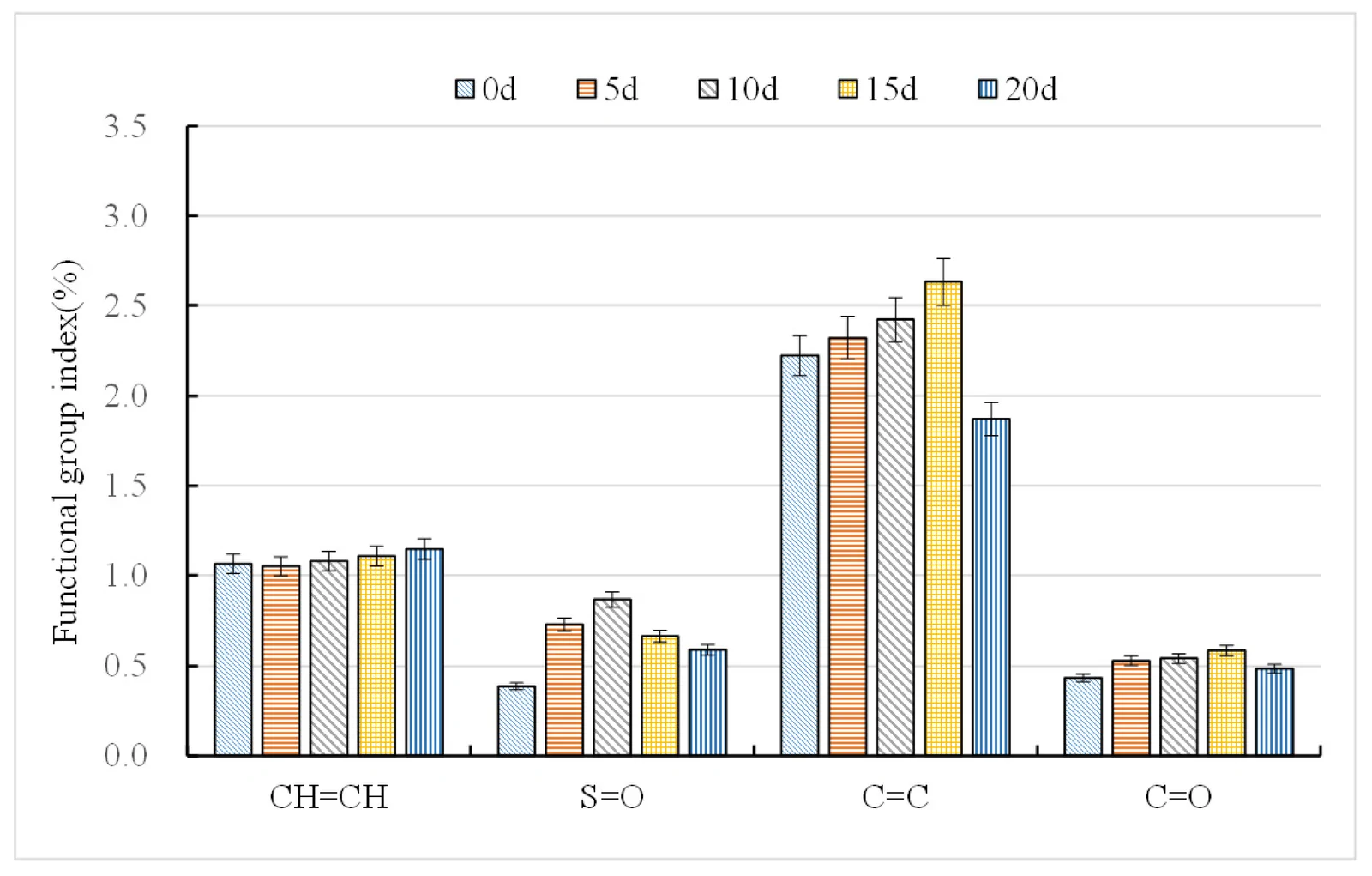
Typical functional group indices of PPA/SBR-MA at different aging durations.

The FTIR absorption spectra of polyphosphoric acid (PPA) and various asphalt samples prior to aging are shown in Figure 8, Figure 9, Figure 10 and Figure 11. As a protic Brønsted acid, PPA contains phospho-oxy acid groups (P=O or P–OH) that can release protons, enabling it to serve as an acid catalyst and readily react with substances abundant in polar components. A comparison of the FTIR spectra of SBR-MA and PPA/SBR-MA in Figure 9 reveals significant differences between the two materials in Regions I–IV. In Region I (3700–3110 cm^−1^), the spectrum of PPA/SBR-MA displays enhanced absorption, which is attributed to the stretching vibration of hydroxyl (–OH) groups, suggesting potential hydrogen-bonding interactions between PPA and polar fractions of asphalt [38]. In Region II, a new peak emerges near 1700 cm^−1^ in the PPA/SBR-MA spectrum, assignable to the stretching vibrations of carbonyl (C=O) or phosphoryl (P=O) groups [39,40]. In Region III, the PPA/SBR-MA spectrum presents a new absorption peak at 1130 cm^−1^, corresponding to the stretching vibration of P–O–C (phosphate ester). Combining this band with the 1700 cm^−1^ peak in Region II, it can be inferred that carbonyl-containing condensation products and phosphate ester structures originate from PPA-catalyzed esterification and dehydration reactions [41]. Previous studies have verified that esterification between PPA and oxygen-active components in asphalt (e.g., phenols) dominates the formation of phosphate ester bonds [40], confirming that phosphoesterification occurs between PPA and asphalt constituents. In Region IV, weak absorption occurs near 510 cm^−1^. Since neat PPA in Figure 8 exhibits no absorption within this wavenumber range, this band is likely related to structural rearrangement of asphalt components induced by PPA.

The above variations in functional groups confirm that PPA incorporation initiates phosphorylation (i.e., phosphate esterification) of base asphalt. Furthermore, PPA acts as an acid catalyst, promoting esterification between asphalt fractions. As documented in previous research, PPA connects asphalt constituents via covalent bonds and promotes the formation of a denser, more stable molecular network [42].

Under the identical coupled aging conditions of intense ultraviolet radiation and large temperature differences adopted in this work, layered ATR-FTIR characterization conducted by our research group in previous studies demonstrated that variations in oxidized components in the lower asphalt layer are faint, while the chemical evolution characteristics of the upper-surface asphalt are far more prominent [9,23]. Accordingly, the upper-surface layer of PPA/SBR-MA was selected for chemical component analysis in this paper. Figure 9 and Figure 10 present the FTIR spectra of the upper-surface binder of PPA/SBR-MA under different aging durations.

From Figure 10, it can be observed that the functional groups on the surface film layer of PPA/SBR-MA exhibit relatively pronounced changes throughout the aging process, particularly at characteristic peaks near 1740 cm^−1^, 3700–3110 cm^−1^, and 1300–1100 cm^−1^. In contrast, other characteristic peaks, such as those associated with aromatic compounds (e.g., at 865 cm^−1^, 810 cm^−1^, and 750 cm^−1^), show no significant variation. During the early stage of aging, the carbonyl peak near 1700 cm^−1^ and the ester peak within the 1300–1100 cm^−1^ range intensify with increasing aging duration; however, both peaks progressively weaken in the later aging stage. The hydroxyl peak in the 3700–3110 cm^−1^ range evolves consistently with the carbonyl peak: hydroxyl absorption emerges after 5 days of aging, strengthens at 10 days, weakens at 15 days, and nearly disappears after 20 days. This overall trend agrees well with prior studies [9,23]. Nevertheless, compared with conventional SBR-MA [9], PPA/SBR-MA exhibits weaker carbonyl absorption at 1700 cm^−1^ and weaker ester absorption in the 1300–1100 cm^−1^ range across all aging durations, indicating that photo-oxidative reactions develop less intensely in PPA/SBR-MA.

To elucidate the aging behavior and anti-aging mechanisms of PPA/SBR-MA, quantitative analyses of sulfoxide (S=O), aromatic (C=C), carbonyl (C=O), and butadiene (CH=CH) indices were conducted. The results are presented in Figure 11 and compared with our previous findings on SBR-MA [9]. The sulfoxide and carbonyl indices of the surface film layer both exhibit an initial increase followed by a decrease, consistent with the evolution pattern observed in SBR-MA. Notably, the sulfoxide index of unaged PPA/SBR-MA is significantly lower than that of SBR-MA, presumably attributable to the esterification reaction between PPA and sulfoxide groups within asphaltene micelles [43].

The progressive weakening and eventual disappearance of hydroxyl peaks (3700–3110 cm^−1^) and ester peaks (1300–1100 cm^−1^) after long-term aging indicate that primary photo-oxidation products undergo polycondensation in the late aging stage and convert into asphaltenes and high-molecular-weight polymers, thereby attenuating the intensity of these characteristic absorption peaks. In contrast, SBR-MA maintains distinct hydroxyl and ester absorption signals throughout aging, suggesting that its surface layer accumulates more oxidation products and develops a thicker surface film. Previous studies have confirmed a crosslinked layer beneath the hardened surface film [23], implying that the PPA/SBR system can rapidly form a dense crosslinked structure and inhibit excessive accumulation of oxidation products. The aromatic C=C index also exhibits an initial increase followed by a decrease, further corroborating the complete evolutionary cycle of the surface film layer during photo-oxidative aging.

Relative to SBR-MA, PPA/SBR-MA exhibits considerably smaller variations in functional group indices, indicating that PPA restrains the formation of polar oxygen-containing groups under coupled intense UV radiation and large temperature variations. The underlying mechanism is that PPA reacts preferentially with active sites to form stable P–O–C bonds and deactivate readily oxidizable functional groups [43]. The butadiene index reflects the relative content of butadiene segments in SBR chains. Upon PPA incorporation, the butadiene index rises, which may stem from PPA promoting uniform dispersion of SBR and enhancing swelling effects, thus improving modification performance. As aging progresses, the butadiene index of PPA/SBR-MA varies far less significantly than that of SBR-MA, demonstrating that PPA effectively mitigates the photo-oxidative degradation of butadiene segments. On the one hand, the crosslinked layer induced by PPA immobilizes SBR particles internally and reduces direct UV exposure; on the other hand, PPA lowers the oxidative reactivity of the system and retards the degradation process. Furthermore, fewer polar oxidation products are generated on the surface of PPA/SBR-MA, substantially weakening the polar adsorption driving force for low-molecular-weight asphaltenes in the underlying layer. Since polar interactions including dipole–dipole coupling and hydrogen bonding serve as key driving forces for the migration of low-molecular-weight asphaltenes toward the surface [24]. The reduction in polar species weakens such adsorption effects and thereby inhibits the upward migration of these asphaltenes.

In summary, PPA interacts with SBR modifier particles to form a spatial network and undergoes esterification with sulfoxide groups in asphaltene micelles, thereby enhancing the crosslinking effect of SBR within asphalt. This inhibits photo-oxidation reactions during aging, reduces the formation rate of polar functional groups (carbonyl and sulfoxide), and suppresses SBR chain degradation, thus enhancing the aging resistance of SBR-MA.

### 3.5. Molecular Size Evolution Analyses

**Figure 12:**
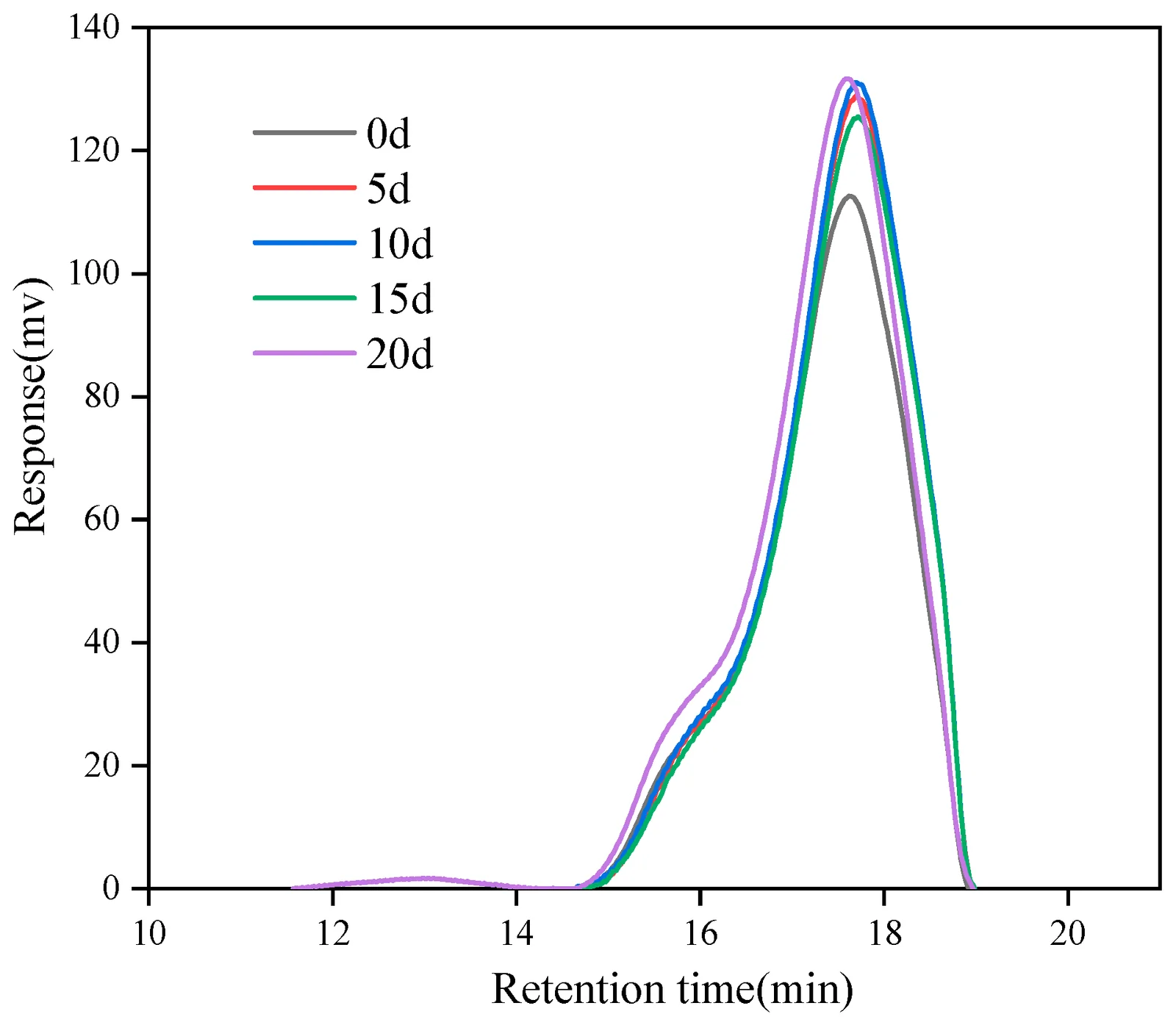
GPC curves of PPA/SBR-MA at different aging durations.

**Figure 13:**
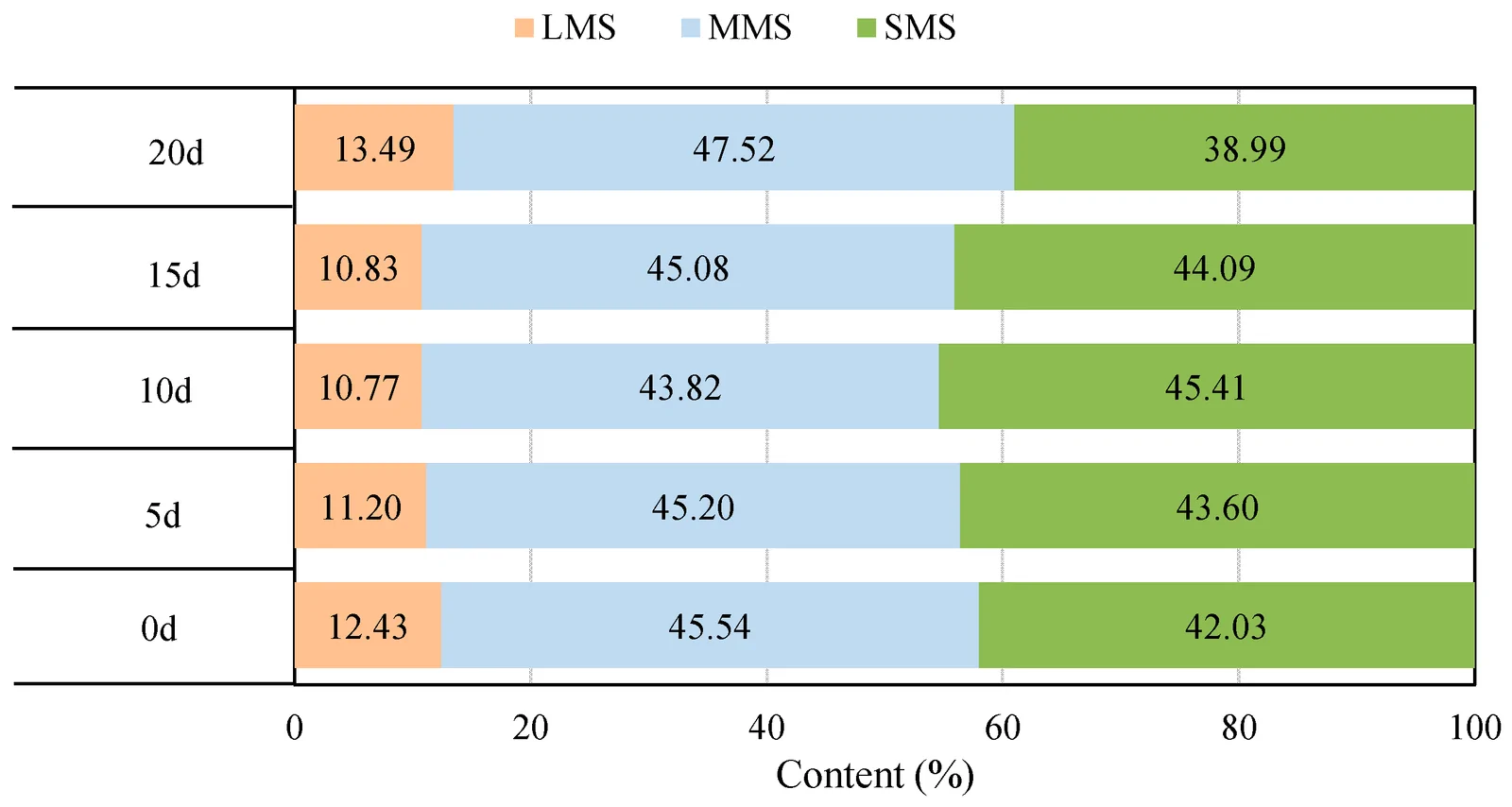
Molecular weight distribution curves of PPA/SBR-MA at different aging durations.

The molecular weight distribution parameters of PPA/SBR-MA are presented in Table 5. The GPC curves and the evolution patterns of large-molecular-size (LMS), medium-molecular-size (MMS), and small-molecular-size (SMS) fractions are shown in Figure 12 and Figure 13, respectively. GPC results indicate that incorporating PPA into SBR-MA increases the number-average molecular weight ($M_{n}$) and weight-average molecular weight ($M_{w}$), while decreasing the polydispersity index (PDI). Concurrently, LMS exhibits an initial increase, MMS decreases markedly, and SMS increases significantly. These observations suggest two parallel pathways through which PPA acts on SBR-MA. The first is a crosslinking pathway. PPA undergoes esterification with SBR particles and polar components in asphalt, generating high-molecular-weight species [44]. FTIR analysis further suggests that PPA can interact with sulfoxide groups within asphaltene micelles, thereby enhancing SBR-related network formation within the asphalt matrix. The second is a dispersion pathway. PPA disrupts medium-sized aggregates, such as asphaltene associations, and converts them into smaller molecular fragments [45], which is consistent with the pronounced reduction in MMS and the increase in SMS. This behavior also aligns with prior reports indicating that PPA induces protonation, disrupts hydrogen bonding, and promotes asphaltene dispersion [46]. These two pathways occur simultaneously. Overall, the dispersion/deaggregation effect predominates, whereas crosslinking homogenizes the molecular weight distribution. This leads to a characteristic distribution pattern, namely a slight increase in LMS, a pronounced reduction in MMS, and a substantial increase in SMS. These observations suggest that PPA undergoes complex chemical interactions with SBR-MA, ultimately giving rise to a more stable dispersed system [18,42].

With increasing aging time, the GPC curve initially shifts toward the low-molecular-weight region, then reverses toward the high-molecular-weight region in the later aging stage. Overall, this trend mirrors that of SBR-MA, but the magnitude of change is markedly smaller, as shown in Figure 12. Component analysis, as presented in Figure 13, reveals that under coupled intense UV radiation and large temperature differences, the LMS fraction in PPA/SBR-MA follows a “decrease-then-increase” pattern. In contrast to SBR-MA, no early-stage LMS rise occurs, suggesting that SBR chain scission is negligible during initial aging. Throughout the entire aging cycle, fewer components in the PPA/SBR-MA system participate in photo-oxidative reactions. The maximum change in LMS after aging is only −13.37%, far lower than the +54.40% observed for SBR-MA, demonstrating that PPA effectively enhances the aging resistance of modified asphalt. The evolution trend of MMS parallels that of LMS, whereas SMS first increases and then decreases. This reflects dominant chain scission and oxidation in the early aging stage, followed by recombination and polymerization of small molecules as well as partial volatilization in the later stage, leading to a net reduction in SMS content.

In summary, under the coupled aging conditions, the molecular weight distribution of PPA/SBR-MA evolves gradually, attributed to PPA’s chemical stabilization effect and crosslinking reinforcement. Correspondingly, the rutting resistance factor exhibits a “decrease–increase” trend, with high-temperature rutting resistance recovering in the late aging stage, indicating excellent resistance to photo-oxidative aging and adaptability to large temperature swings. In contrast, SBR-MA is prone to photo-oxidative degradation. Its rutting resistance continues to deteriorate in the late aging stage, accompanied by accelerated volatilization of small molecules, rendering it inadequate for long-term pavement durability in regions subject to intense UV radiation and wide temperature variations. The evolution patterns of molecular weight distribution for both asphalt binders correlate well with the variation characteristics of the rutting resistance factor, further validating the proposed mechanisms and reinforcing the conclusion that PPA effectively retards the aging degradation of SBR-MA.

### 3.6. Microstructural Transformations

#### 3.6.1. Microstructural Characterization

**Figure 14:**
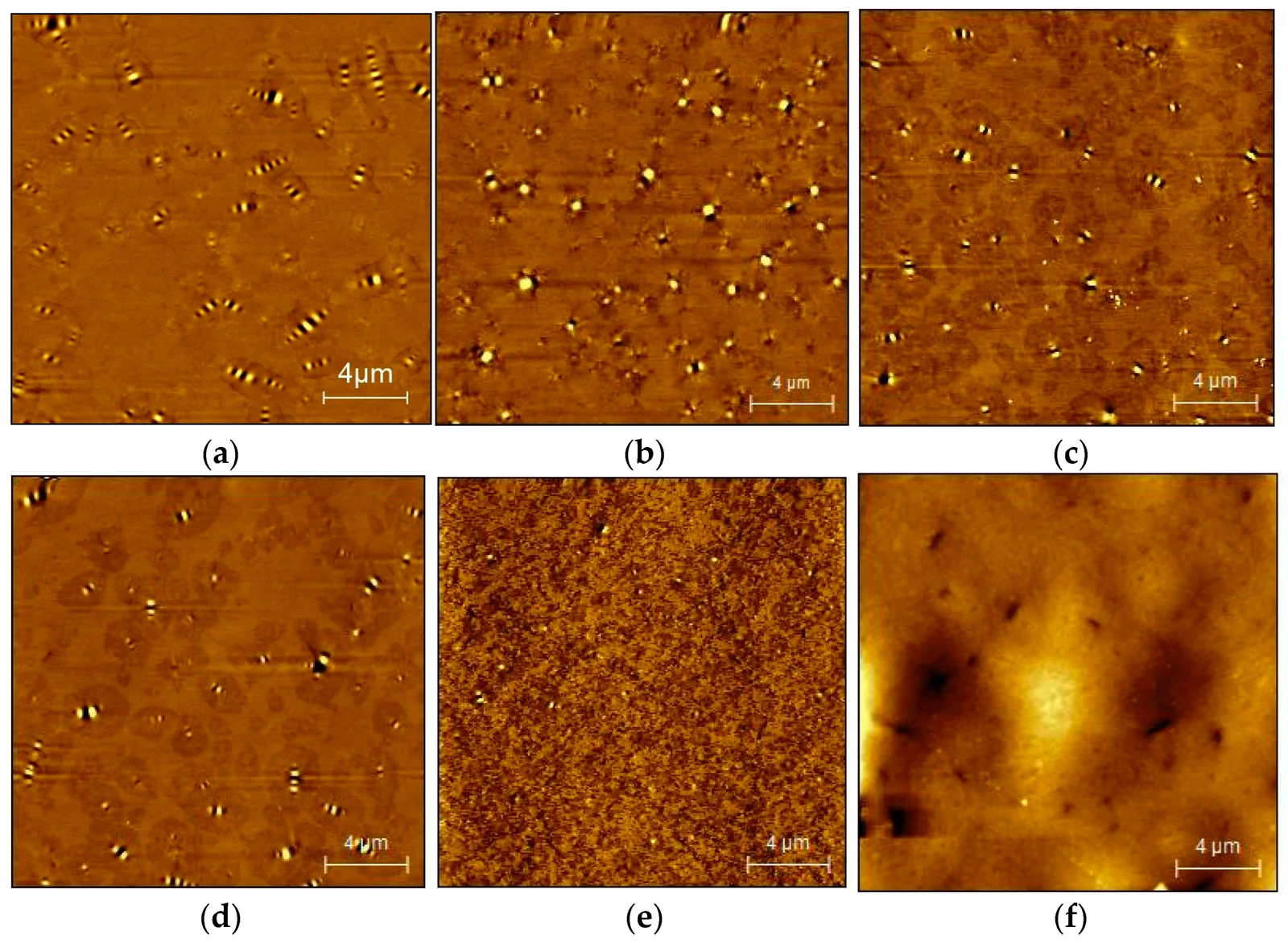
Micro-surface morphology of PPA/SBR-MA at different aging durations: (**a**) 0 d, (**b**) 5 d, (**c**) 10 d, (**d**) 15 d, (**e**) 20 d, and (**f**) 20 d (surface film layer).

As shown in Figure 14, the PPA/SBR-MA exhibits the re-emergence of the bee structure, indicating that the addition of PPA drives the asphalt system toward a colloidal structure resembling that of neat base asphalt. This finding provides a plausible mechanistic interpretation for the improved high-temperature storage stability of SBR-MA upon PPA incorporation, which stems from enhanced compatibility between SBR and the asphalt matrix [17,47]. Figure 14 presents the two-dimensional microstructures of the bulk asphalt layer for PPA/SBR samples at different aging durations, as well as the surface thin-film layer after 20 days of aging.

Previous studies have confirmed that the bee structure in neat base asphalt originates from the ordered association of asphaltenes [9]. After incorporating SBR into base asphalt, the polymer forms a three-dimensional spatial network to break up asphaltene aggregates and disperse them uniformly. The original ordered aggregation state is destroyed, and the bee structure disappears accordingly [23]. In contrast, when PPA is added to SBR-MA, PPA acts as a Brønsted acid and chemically reacts with polar functional groups in asphalt via esterification. This increases the overall polarity of the system, promoting re-aggregation and ordered assembly of asphaltenes; consequently, the bee structure re-emerges under AFM imaging. This “disappearance → reappearance” evolution pattern suggests that PPA induces the formation of a new crosslinked network or an ordered phase structure.

Figure 14 also shows that in the bulk asphalt layer of PPA/SBR-MA, both the size and population of the bee structure gradually diminish with increasing aging duration. This evolutionary trend is comparable to that of SBR-MA, yet notable distinctions exist in the two-dimensional microstructures and phase morphology between the two binders. Upon incorporation of PPA, the disaggregation rate of macromolecular asphaltenes is retarded, and the extent of disaggregation is significantly suppressed. Small bee structures remain detectable within the bulk layer of PPA/SBR-MA even after 20 days of aging. The retention of such bee structures implies that the PPA-induced crosslinked network and ordered phase structure exhibit adequate stability against the synergistic destructive effects of UV irradiation and thermal oxidation during aging. This observation agrees well with prior research [48], which stated that improved colloidal stability can effectively retard microstructural degradation of modified asphalt under harsh environmental conditions.

For the surface thin-film layer after 20 days of aging, obvious attenuation of dark aggregative phases is observed in PPA/SBR-MA relative to SBR-MA. These dark aggregative phases generally correspond to accumulated polar oxidation products such as carbonyl and sulfoxide compounds formed throughout aging. Accordingly, the evident reduction in these phases directly indicates less generation of aging by-products within the PPA/SBR system. Combined with the evolutionary characteristics of the bee structure described above, it can be concluded that PPA strengthens the colloidal framework to elevate asphalt high-temperature stability; meanwhile, it improves aging resistance by slowing asphaltene disaggregation and inhibiting the formation of polar oxidation products. This mechanistic insight provides a reasonable interpretation for the outstanding performance of PPA/SBR-MA under coupled intense UV irradiation and large temperature fluctuations, typical service conditions for pavements in high-altitude and desert regions.

#### 3.6.2. Micromechanical Analysis

In this section, the Young’s modulus and adhesion force measured by AFM were used to characterize the evolution of the micromechanical properties of SBR-MA and PPA/SBR-MA during aging. Both parameters were derived from the AFM force–displacement curves. Specifically, the adhesion force was calculated as the force differential between the contact and detachment points, while Young’s modulus was determined using the DMT model in contact mechanics to fit the probe’s retraction process. Detailed procedures are described in our previous studies [7,23].

**Figure 15:**
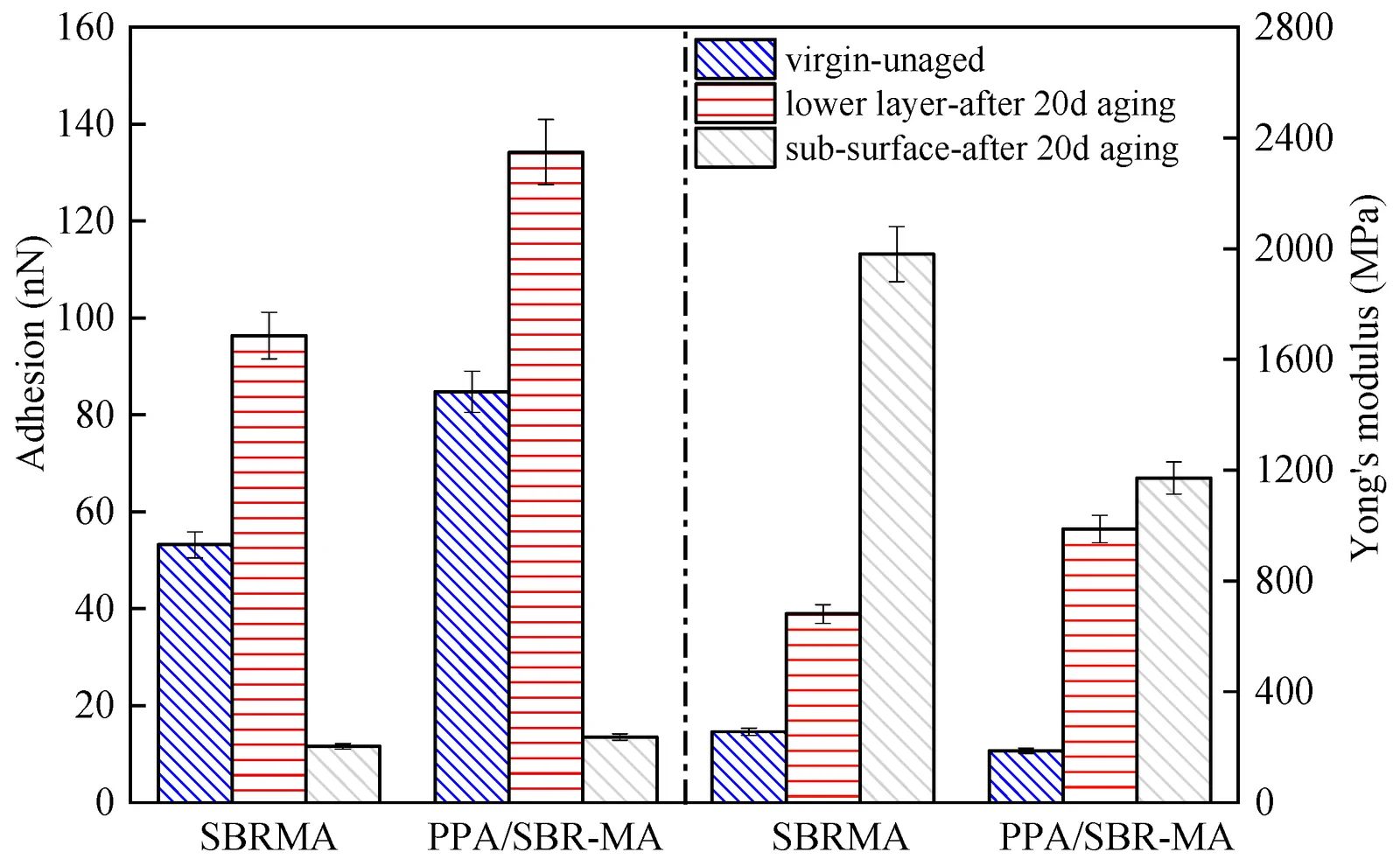
Nanoscale adhesion forces of asphalts before and after 20 d of aging.

**Figure 16:**
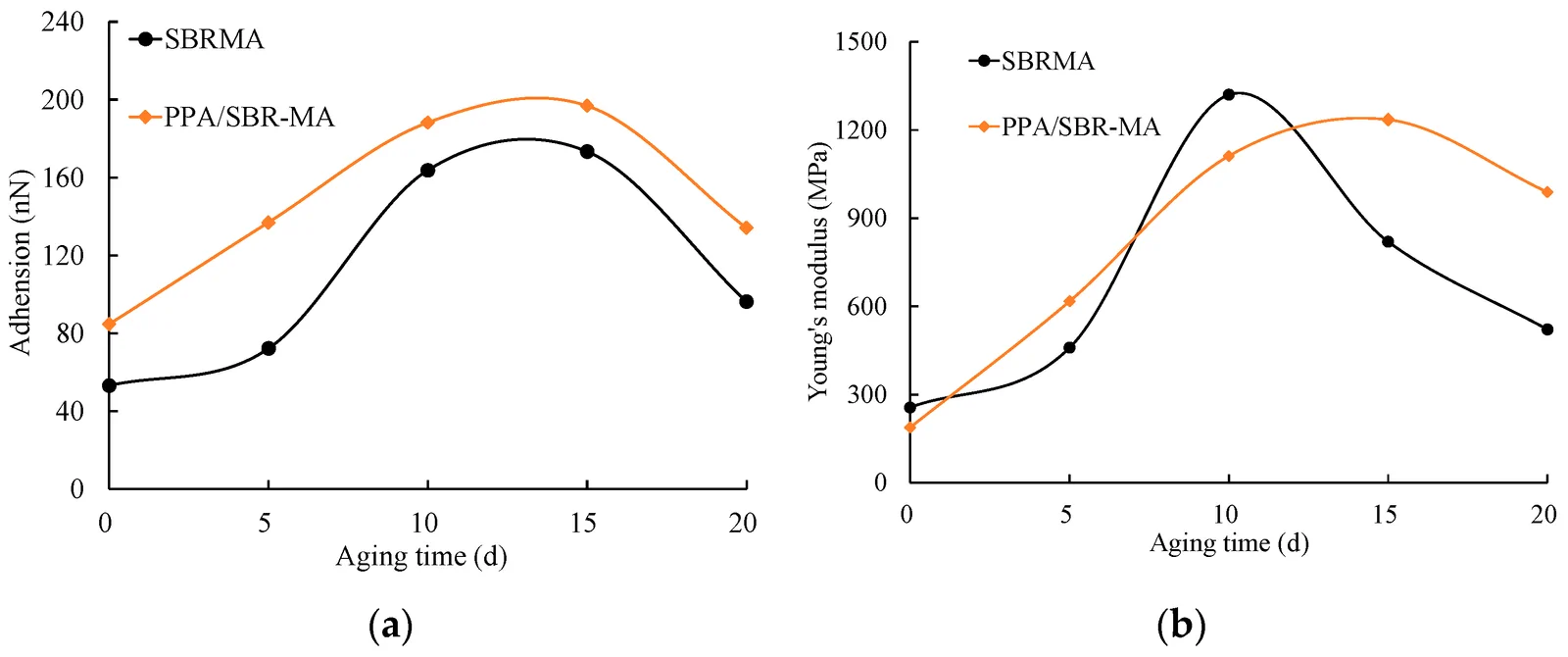
Nanoscale adhesion force (**a**) and Young’s modulus (**b**) of the underlying asphalt.

This section examines the surface micromechanical properties of PPA/SBR-MA. Figure 15 and Figure 16 present the micromechanical results of SBR-modified and PPA/SBR-MA before aging and after 20 days of aging. As shown in Figure 15, the nanoscale adhesion force of SBR-MA increases markedly upon incorporation of polyphosphoric acid. This phenomenon indicates that PPA effectively enhances the tackiness of the asphalt binder. After 20 days of aging, the nanoscale adhesion force of the top surface film layer of PPA/SBR-MA becomes comparable to that of SBR-MA. It reflects a sharp decline in surface adhesion during the middle and late stages of aging. This implies that the physical properties of aged surface films tend to be consistent regardless of binder formulations, resulting in uniformly low nanoscale adhesion forces for different modified asphalt systems. In contrast, the nanoscale adhesion force of the bulk asphalt layer remains highly dependent on binder composition. After 20 days of aging, the bulk layer of PPA/SBR-MA exhibits a significantly higher nanoscale adhesion force compared with its unaged state, with a substantially greater increment magnitude than that of pure SBR-MA.

In addition, PPA addition only induces marginal variation in the Young’s modulus of SBR-MA. The Young’s modulus of PPA/SBR-MA decreases slightly relative to SBR-MA, which suggests an optimized molecular weight distribution and improved phase compatibility. After 20 days of aging, both the surface film layer and bulk asphalt layer show substantial increases in Young’s modulus. Nevertheless, the increment of surface Young’s modulus is significantly attenuated for PPA/SBR-MA compared with SBR-MA. To quantitatively characterize the gradient aging behavior, the modulus ratio, defined as the ratio of surface film Young’s modulus to bulk asphalt Young’s modulus, is calculated according to a previously reported method [23]. The modulus ratio decreases from 3.8 for SBR-MA to 1.2 for PPA/SBR-MA, corresponding to a reduction of 68.4%. This result indicates that PPA effectively narrows the modulus mismatch between surface and bulk layers. The optimized mechanical gradient mitigates the gradient aging effect of SBR-MA and reduces the propensity for surface cracking. This micromechanical evidence reasonably explains the delayed initiation and milder degree of surface cracking of PPA/SBR-MA, which is consistent with the aforementioned surface morphology analysis results.

To further clarify the superior crack resistance of PPA/SBR-MA, comparative analyses of nanoscale adhesion force and Young’s modulus at different aging durations are performed based on Figure 16. The nanoscale adhesion force of PPA/SBR-MA remains consistently higher than that of SBR-MA throughout the entire aging process. Relative to the corresponding unaged samples, the maximum increment of nanoscale adhesion force reaches 132.3% for PPA/SBR-MA and 226.0% for SBR-MA. This comparison indicates that SBR-MA suffers more severe mechanical degradation under the coupled effects of intense UV radiation and thermal cycling. In the late aging stage, PPA/SBR-MA possesses higher Young’s modulus values than SBR-MA. The improved mechanical uniformity reduces the modulus differential between surface and bulk layers and lowers the modulus ratio. This reduced mechanical heterogeneity fundamentally lowers the surface cracking risk of aged modified asphalt.

## 4. Conclusions

In this study, PPA was incorporated into SBR-MA for composite modification to address its insufficient high-temperature deformation resistance and poor anti-aging performance during late-stage aging, thereby improving its service performance under coupled intense ultraviolet radiation and large temperature differences. Through macroscopic performance tests and microscopic structural characterization, the evolution of performance and microstructure during aging was systematically investigated, and the intrinsic anti-aging mechanism was further clarified. The main research conclusions are summarized as follows.

(1) Macroscopic surface morphology results demonstrated that PPA/SBR-MA exhibited superior surface stability compared with conventional SBR-MA under coupled aging conditions. The incorporation of PPA effectively slowed the surface darkening rate, delayed the initiation of network-like microcracks, and significantly reduced the cracking severity, indicating a milder and more gradual aging progression throughout the aging process.
(2) Both the high-temperature performance (rutting factor G*/sinδ) and fatigue life of PPA/SBR-MA showed a three-stage evolution, in contrast to the single-peak pattern of SBR-MA. Specifically, the rutting factor increased rapidly from 0 d to 10 d, slightly declined at 15 d, and then increased again at 20 d, while fatigue life followed an increase–decrease–reincrease trend. In the late stage, the stress–strain curve exhibited a broader peak width, indicating improved toughness and fatigue resistance. These results suggest that PPA delays the onset of performance deterioration and induces a late-stage re-enhancement effect, thereby compensating for the deficiencies of SBR-MA in rutting resistance and fatigue durability under long-term intense UV irradiation and large temperature differences.
(3) Chemical and molecular analyses revealed that PPA esterified polar components in asphalt, suggesting the formation of stable P–O–C bonds and reducing carbonyl index ($I_{C=O}$) variation. Meanwhile, PPA appeared to induce a dense cross-linked network beneath the surface film, likely retarding SBR chain degradation, as reflected by the reduced variation in butadienyl index ($I_{CH=CH}$). This network also appeared to inhibit the accumulation of oxidation products and weakened the polar adsorption driving force of small-molecule-weight asphaltenes, thereby suppressing their upward migration. Gel permeation chromatography (GPC) further supported a dual cross-linking dispersion effect, optimizing the molecular weight distribution and reducing the polydispersity index (PDI). The improved structural homogeneity may further retard molecular chain degradation, contributing to the enhanced anti-aging performance of the composite-modified asphalt.
(4) AFM results indicated that the incorporation of PPA improved the uniformity of the asphalt matrix and enhanced the compatibility between SBR and asphalt. After 20 days of aging, distinct bee structures were still observed in PPA/SBR-MA, and the nanoscale modulus ratio (defined as the ratio of surface film modulus to bulk asphalt modulus) decreased by 68.4% compared with aged SBR-MA. Combined with macroscopic surface analysis, these results indicated that PPA effectively alleviated the gradient aging of SBR-MA, reduced depth-wise material heterogeneity, and thereby improved cracking resistance. Therefore, PPA/SBR-MA is expected to exhibit better applicability than conventional SBR-MA in pavement engineering in high-altitude cold regions.

### Future Research Work

This study demonstrates that PPA/SBR-MA exhibits significantly enhanced aging resistance compared to SBR-MA under coupled aging induced by intense UV radiation and large temperature differences. Accordingly, PPA/SBR-MA is recommended for application in high-altitude cold regions (e.g., the Tibetan Plateau). However, several limitations should be noted. The effectiveness of the modification depends on the PPA dosage and the source of the base asphalt binder; therefore, its applicability should be verified for specific combinations of base asphalt sources and PPA dosages. In addition, its suitability for other climatic regions or different pavement types has not yet been evaluated. Future research will conduct field aging validation and long-term pavement performance studies of PPA/SBR-MA mixtures in the Tibetan Plateau. Furthermore, the PPA dosage and its combination with other modifiers will be optimized to further broaden the applicability of PPA/SBR-MA. Moreover, life-cycle cost analysis for large-scale engineering applications will be performed, and the performance of PPA/SBR-MA in other extreme environments will be evaluated to further define its applicability boundaries.

## Figures and Tables

**Table 1:** Technical specifications of main materials.

Material	Technical Specifications
SBR	White powder; bound styrene content 23.0%; tensile strength 25 MPa; elongation at break 378%; volatile content 0.6%
PPA	Colorless and transparent viscous liquid; H_3_PO_4_ content 115.2%; P_2_O_5_ content 85.3%

**Table 2:** Basic physical properties of asphalt binders.

Asphalt	Penetration (25 °C, 5 s, 100 g)/0.1 mm	Ring-and-Ball Softening Point (25 °C)	Ductility (5 °C)/cm	High-Temperature Storage Stability (°C)	Density (g/cm^3^)
Shell 90	88.4	47.7	13.5	0.4	1.030
SBR asphalt	76.0	52.3	>100	0.9	1.031
PPA/SBR asphalt	68.5	56.5	>100	0.4	1.034

**Table 3:** Definitions of parameters.

Parameter	Wavenumber (cm^−1^)	Peak Area
Butadiene	966	$A_{966{\mathrm{cm}}^{-1}}$
Sulfoxide	1030	$A_{1030{\mathrm{cm}}^{-1}}$
Aromatic hydrocarbon	1600	$A_{1600{\mathrm{cm}}^{-1}}$
Carbonyl	1700	$A_{1700{\mathrm{cm}}^{-1}}$
All characteristic peaks within the wavenumber range	450–4000	$\sum A_{450{\mathrm{cm}}^{-1}–4000{\mathrm{cm}}^{-1}}$

**Table 4:** Peak stress and strain of each curve.

Asphalt Type	0 d		5 d		10 d		15 d		20 d	
	Peak Stress (kPa)	Strain (%)	Peak Stress (kPa)	Strain (%)	Peak Stress (kPa)	Strain (%)	Peak Stress (kPa)	Strain (%)	Peak Stress (kPa)	Strain (%)
SBRMA	273.2	13.49	303.6	12.14	343.9	12.23	337.6	12.34	309.8	11.93
PPA/SBR-MA	242.3	15.12	303.0	14.54	318.1	14.83	304.2	14.26	294.4	14.93

**Table 5:** Molecular weight and polydispersity index of PPA/SBR-MA and SBR-MA.

Asphalt Sample	$M_{n}$	$M_{w}$	PDI
SBR-MA	780	1967	2.601
PPA/SBR-MA	800	2063	2.579

## Data Availability

The data presented in this study are available in the article. Further inquiries can be directed to the corresponding author or the first author.
